# The kynurenine pathway storm in epilepsy: mechanisms and therapeutic implications

**DOI:** 10.1186/s42494-026-00250-w

**Published:** 2026-05-01

**Authors:** Yuxuan He, Jing Meng, Wen Luo, Mingxing Yu, Nana Zhang, Chunyan Chen, Liang Yu

**Affiliations:** 1 School of Medicine, University of Electronic Science and Technology of China, Chengdu, 610054 China; 2 Department of Neurology, Sichuan Provincial People’s Hospital, School of Medicine, University of Electronic Science and Technology of China, Chengdu, 610072 China; 3 Chengdu University of Traditional Chinese Medicine, Chengdu, 610075 China

**Keywords:** Epilepsy, Kynurenine pathway, Neuroinflammation, Oxidative stress, Microbiome–gut–brain axis, Epilepsy combined with depression

## Abstract

The kynurenine pathway (KP) is the main route for tryptophan (TRP) degradation and plays a crucial role in neuroinflammation, oxidative stress and neurotransmission, which holds significant clinical significance. The dysregulation of this pathway is closely related to neurological disorders such as epilepsy, and its metabolic products can promote the occurrence of epileptic seizures and comorbid depression. This review aims to clarify the complex mechanisms of the KP in the occurrence and development of epilepsy and to explore its potential as a therapeutic target for epilepsy and comorbid depression. This is a narrative review and synthesis of the current literature. We reviewed animal experiments and clinical studies, elaborating in detail on how metabolites of the KP and their key enzymes function in the context of epilepsy by regulating neuroinflammation, oxidative stress, glutamatergic signaling, and the gut-brain axis. We also explored the interaction between antiseizure medications (ASMs) and the KP, and evaluated the potential value of targeting key enzymes (such as indoleamine 2,3-dioxygenase, IDO) as a new therapeutic strategy for epilepsy. This review particularly focuses on the promoting effect of KP imbalance on comorbid depression, clarifying how IDO-mediated TRP metabolism changes constitute a common mechanism basis, jointly leading to the occurrence of epilepsy and depression-like behaviors. The occurrence and development of epilepsy are closely related to the imbalance of the KP, specifically manifested as a decrease in kynurenic acid (KYNA) level and an increase in quinolinic acid (QA) level. The IDO-mediated shift of TRP metabolism towards the KP is established as a critical mechanism underlying depression comorbidity in epilepsy. Therapeutic modulation of this pathway, through targeting key enzymes like IDO and restoring the KYNA/QA balance, presents a viable strategy for improving the cerebral microenvironment. This approach holds promise for enhancing seizure control, counteracting drug resistance, and concurrently alleviating comorbid depressive symptoms.

**Graphical Abstract:**

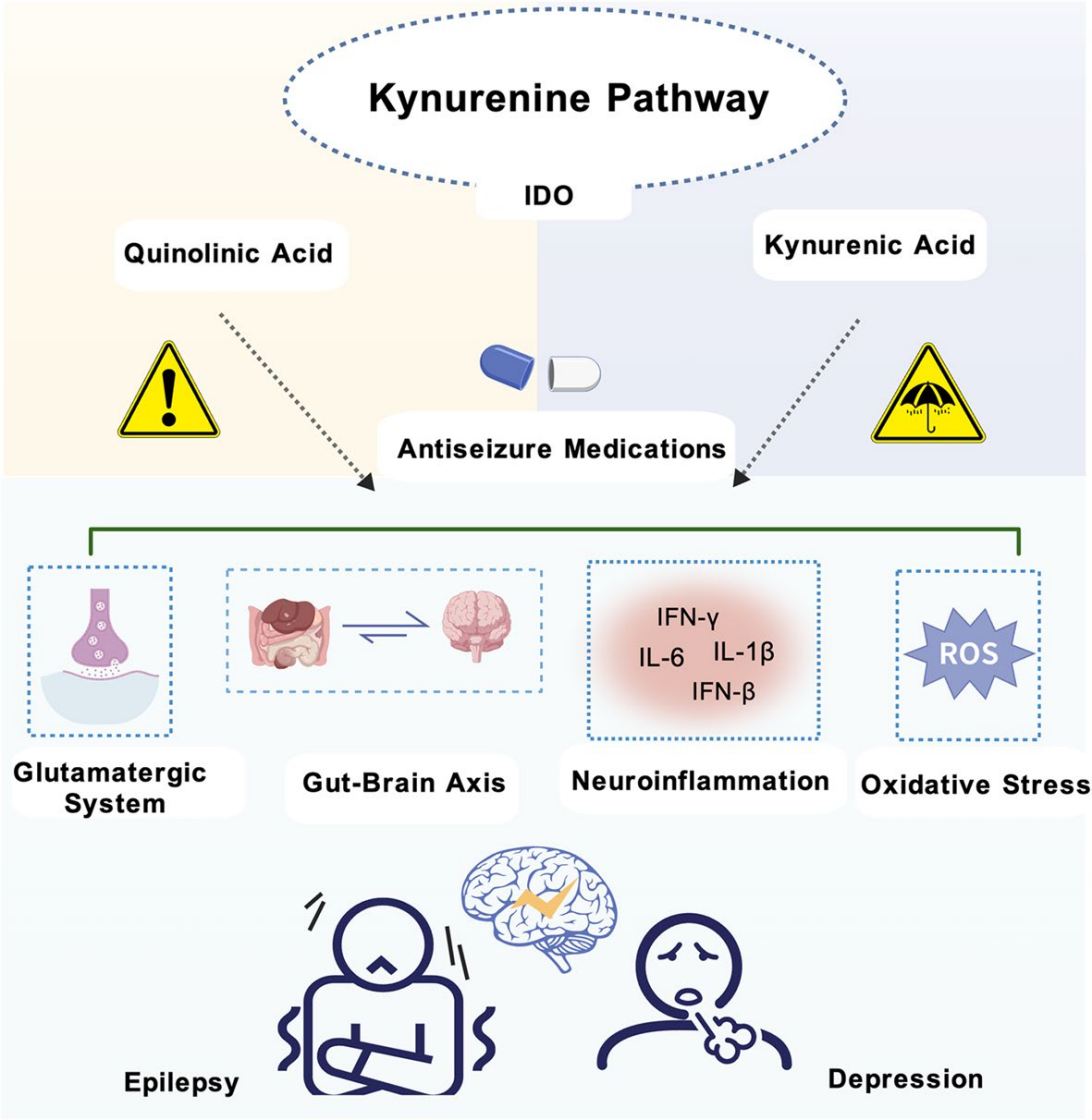

## Background

Epilepsy is a neurological disorder characterized by transient and synchronous abnormal discharges of brain neurons [1]. Recurrent epileptic seizures not only seriously affect the quality of life of patients, but also extend their harm beyond the neurological field to the psychological, cognitive and somatic comorbidity levels. About 32% of epilepsy patients are troubled by depression, a proportion that is particularly significant in this group [2]. In addition, patients may also face adverse reactions caused by treatment, including central nervous system reactions (CNS) (such as drowsiness, dizziness, headache), rashes, gastrointestinal reactions (such as nausea, vomiting), and effects on the metabolic and endocrine systems [3]. Therefore, clarifying the novel pathophysiological mechanisms underlying the occurrence and development of epilepsy is a key step towards developing safer and more effective treatment methods.

The kynurenine pathway (KP) is the main metabolic route for tryptophan (TRP) degradation in the human body and has received considerable attention in the field of CNS. Under the stimulation of inflammatory factors, indoleamine 2,3-dioxygenase (IDO) catalyzes the conversion of TRP to kynurenine (KYN), thereby initiating this metabolic pathway. One of the main metabolites, quinolinic acid (QA), functions primarily as an N-Methyl-D-aspartate (NMDA) receptor agonist, leading to calcium overload, mitochondrial dysfunction, and increased generation of reactive oxygen species (ROS) [4–6]. This imbalance further spreads to the homeostasis of the glutamatergic and γ-aminobutyric acid (GABA) systems, intensifying neuroinflammatory responses and ultimately leading to neuronal hyperexcitability [4, 7]. The neuroinflammation, oxidative stress, and excitability enhancement induced by QA form a mutually reinforcing vicious cycle [8], jointly promoting the occurrence and development of epilepsy. In contrast, kynurenic acid (KYNA) effectively counteracts the effects of QA through multiple mechanisms, such as antagonizing NMDA receptors and activating G protein-coupled receptor 35 (GPR35) [9, 10].

To improve the management of epileptic seizures, researchers have been investigating novel targets for intervention. Studies indicate that gut microbiota (GM) contribute to the pathogenesis of epilepsy through neurological, immunological, endocrine, and metabolic pathways, in addition to upward pathways mediated by the intestine and blood–brain barriers (BBB) [11]. Following an epileptic seizure, the down-regulation pathways mediated by the hypothalamic–pituitary–adrenal axis and the autonomic nervous system could result in modifications in the GM associated with epilepsy, which may then trigger another seizure through upward signaling mechanisms [12]. After an epileptic seizure, the body is in a state of low-grade systemic inflammation [13]. Local intestinal inflammation can activate IDO, thereby initiating the KP. Alterations in the gut microbiota can influence KP metabolism through immune and endocrine pathways. The large number of metabolites such as QA generated under this condition can pass through the damaged BBB and enter the CNS, further intensifying neuroinflammation and oxidative stress, and lowering the threshold for epileptic seizures [14, 15]. KYN and KYNA modulate intestinal immune responses and inflammatory processes through interactions with aryl hydrocarbon receptor (AhR) and GPR35, and subsequently transmit signaling information to the CNS.

The KP not only participates in the occurrence of epilepsy through metabolite-mediated neurotoxicity and neuroinflammation, but also becomes an important mechanism promoting comorbid depression due to its characteristic of disrupting the balance of TRP metabolism. TRP is a common metabolic precursor for the synthesis of serotonin (5-HT) and entry into the KP. Under pathological conditions, inflammatory factors such as IFN-γ can induce the activation of IDO, driving TRP metabolism towards the KP [16]. This shift leads to an increase in the production of QA and 3-HK, while reducing 5-HT synthesis. The further neuroinflammation stimulated by QA and 3-HK forms a positive feedback loop that promotes the continuous activation of IDO, ultimately maintaining 5-HT levels at a low state [17].What is more complicated is that traditional antiseizure medications (ASMs) such as carbamazepine, as potent inducers of liver enzyme P450, can significantly reduce the blood concentration of concomitantly used antidepressants (such as sertraline), thereby weakening their therapeutic effect [18]. Thus, inhibiting IDO constitutes a safe and effective therapeutic strategy against epilepsy and comorbid depression.

IDO inhibitors have shown potential in the treatment of epilepsy, especially for inflammation-related types of epilepsy [19]. After IDO activates the KP, kynurenine monooxygenase (KMO) regulates the flow of metabolic intermediates towards the QA synthesis branch or the KYAN synthesis branch. Negative regulation of the activity of KMO can effectively reduce the accumulation of QA, break the vicious cycle of neuroinflammation-oxidative stress in epilepsy, and ultimately lower the frequency and severity of epileptic seizures [7]. Research indicates that existing ASMs may exert their therapeutic effects by regulating the imbalance of KP. For instance, levetiracetam can promote the production of anticonvulsant metabolites KYNA and xanthurenic acid (XA) by astrocytes, while simultaneously inhibiting the release of proconvulsant metabolites cinnabarinic acid (CA) and QA. Notably, carbamazepine may be metabolized into QA within the BBB, offering a new perspective on the mechanism of drug resistance. In conclusion, regulating the balance of the KP may be a common mechanism of action for multiple ASMs. Targeting the key enzymes of this pathway is expected to become a new strategy for adjunctive epilepsy treatment and provide a new direction for clarifying the mechanism of drug resistance.

## KP in the brain

TRP can be metabolized into various compounds, with 5-HT being the most notable. In fact, approximately 95% of TRP is degraded through KP [20], eventually generating neuroactive metabolites including QA and KYNA [21]. The formation of the primary intermediate metabolite, KYN, represents a rate-limiting enzymatic step in the KP cascade [22]. The reaction is catalyzed by IDO in extrahepatic locations (such as in the brain or immune system) or tryptophan 2,3-dioxygenase (TDO) mostly within the liver. The overall functional outcome of the KP depends on the relative catalytic activities of KMO and kynurenine aminotransferase (KAT) on KYN [21].

### The main route of KP: leading to QA synthesis

**Fig. 1:**
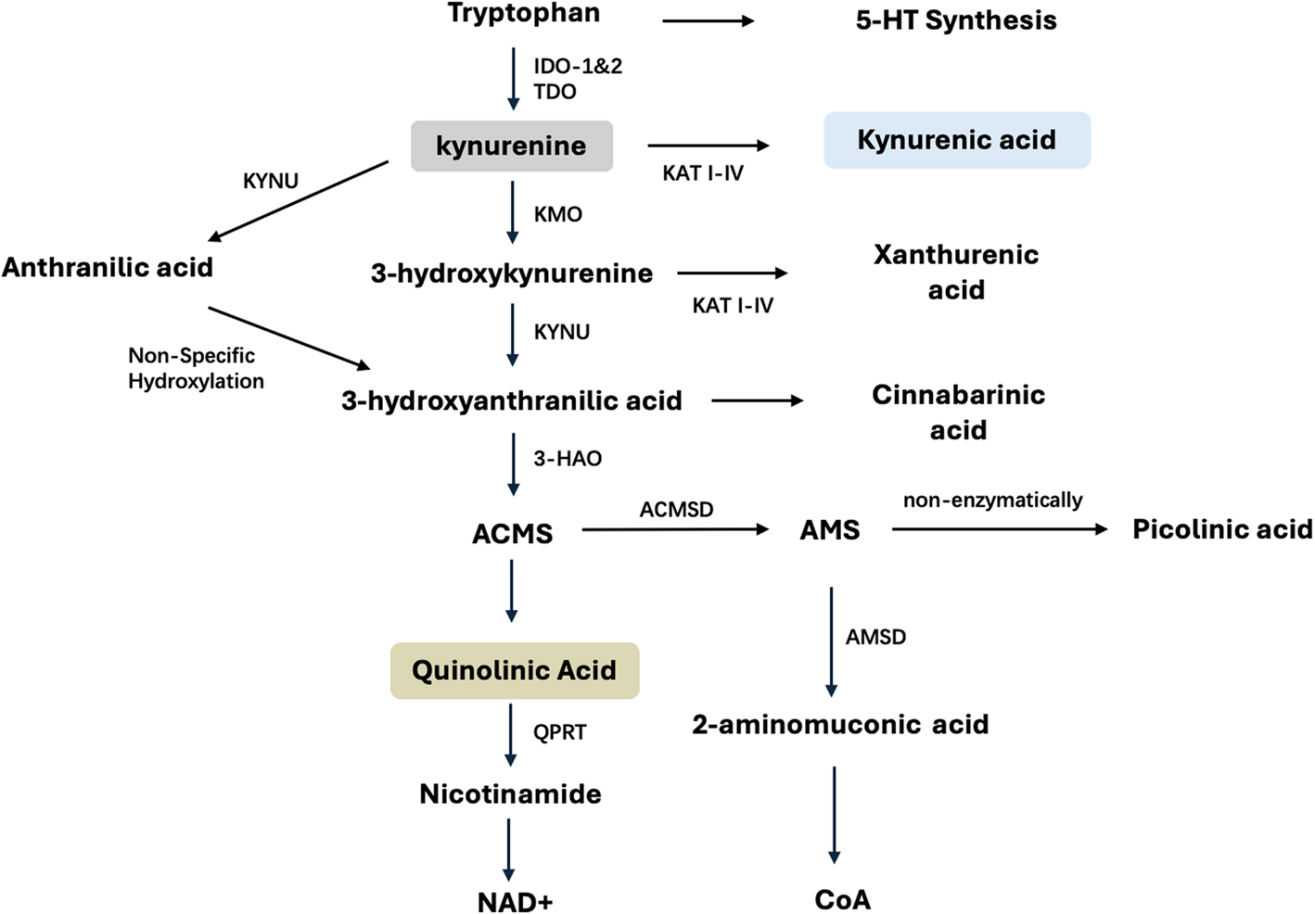
The major metabolites and enzymes of KP. The KP mainly proceeds along a single main line. Under the catalysis of *IDO* or *TDO*, *KMO*, *KYNU*, *3-HAO* and *quinolinate phosphoribosyltransferase* successively, TRP is converted into KYN, 3-HK, 3HAA, ACMS, QA, NAD + in sequence. The four branches of KP:1) KYN is converted into KYNA by *KAT I-IV*; 2) 3-HK is converted into XA by KAT I-IV; 3) KYNU converts KYN into AA; AA is then transformed into 3-HAA; 3-HAA is ultimately a precursor for CA. 4) 3-HAA is catalyzed by *ACMSD* to form 2-amino-3-muconic acid-6-semialdehyde (AMS), which then non-enzymatically generates PIC or is metabolized by *AMSD* to 2-aminomuconic acid, and finally 2-aminomuconic acid is degraded into acetyl coenzyme A

KP metabolism mainly proceeds along a single main line (see Fig. 1). Under physiological conditions, KYN is predominantly converted into 3-hydroxykynurenine (3-HK) through the enzymatic activity of KMO. Subsequently, 3-HK is hydrolyzed by kynureninase (KYNU) into 3-hydroxyanthranilic acid (3HAA). 3-HK is a pro-oxidative, pro-inflammatory and neurotoxic metabolite, and its toxic mechanism is mainly related to oxidative stress [23]. However, 3-HK exhibits a dual role in redox processes, with its ultimate effect depends on the cellular microenvironment. Under physiological conditions, 3-HK mainly exerts an antioxidant effect, but in states of inflammation, oxidative stress or at high concentrations, it tends to promote oxidative stress [24]. Both 3-HK and 3-HAA concentration-dependently reduce the mitochondrial membrane potential in rat cortical astrocytes and striatal tissues, indicating that they directly impair mitochondrial function and ultimately cause metabolic disturbances in cells [25].

3HAA is subsequently converted to α-amino-α-carboxymuconic-ω-semialdehyde (ACMS) via 3-hydroxyanthranilic acid 3,4-dioxygenase (3HAO) [26]. ACMS exhibits instability and can spontaneously convert to QA at physiological concentrations [27]. QA, a primary cytotoxic metabolite in the KP, is produced predominantly by activated microglia. Elevated concentrations of QA are associated with axonal degeneration. In organotypic cultures of rat cortical-striatal tissue, prolonged exposure to low concentrations of QA (100 nM) for 7 weeks can induce excitotoxic injury [21]. As early as the last century, intraventricular administration of QA was observed to trigger convulsions in mice. QA has been demonstrated to induce degeneration of hippocampal pyramidal neurons, particularly in the CA3 and CA1 regions. A dose of 3 nM was sufficient to induce electroencephalogram changes in mice, characterized by recurrent high-voltage sharp waves, whereas a higher dosage of 120 nM is required to provoke epileptic seizures [28].

### Four branche routes of KP

**Fig. 2:**
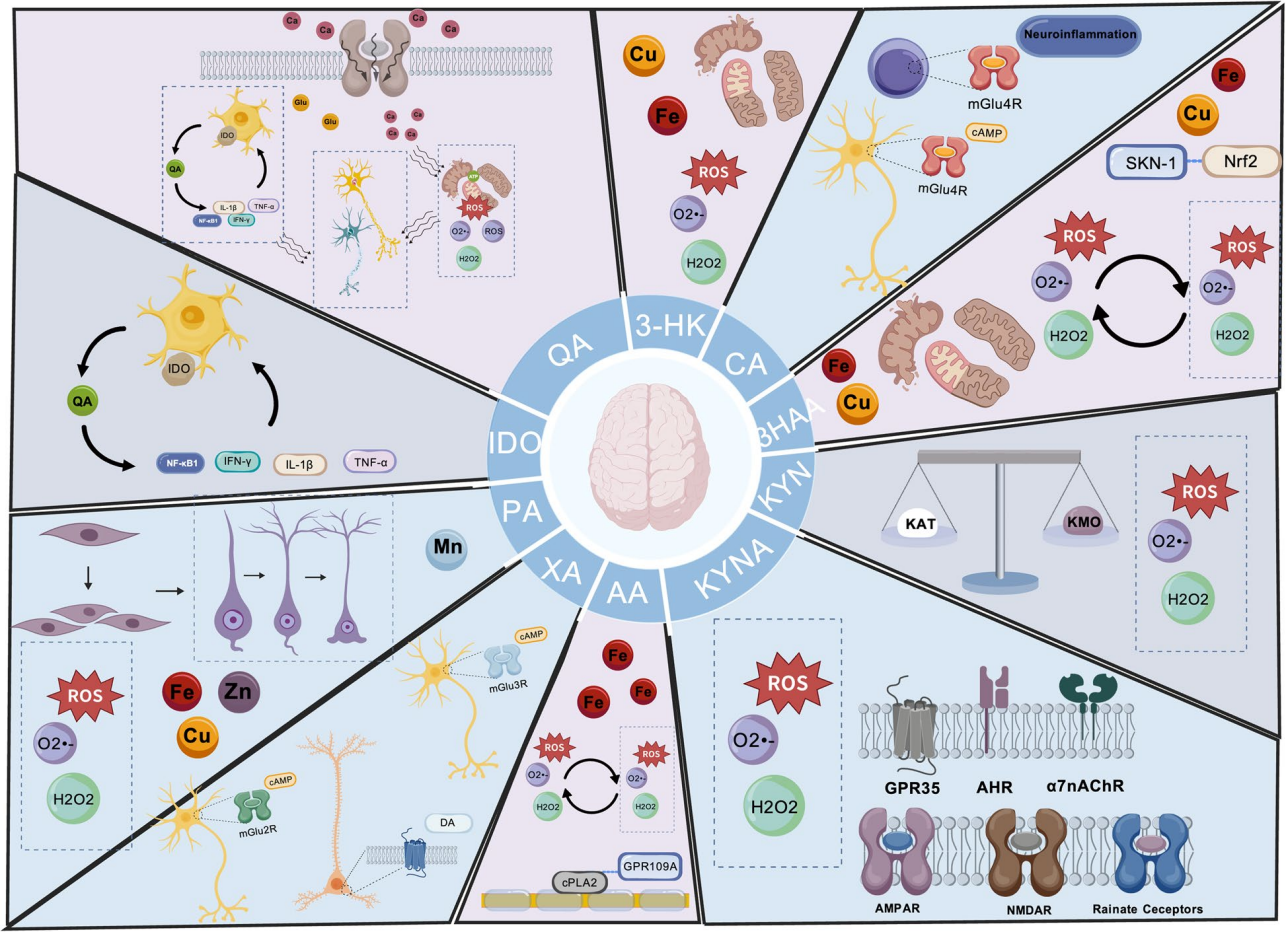
The primary roles of various kynurenine metabolites in the nervous system (Created with BioGDP.com [35]) QA: activates NMDAR, leading to calcium influx, intracellular calcium overload, mitochondrial damage, increased ROS production, release of pro-inflammatory factors, and ultimately neuronal death; 3-HK: increases ROS production, damages mitochondria; CA: activates mGluR4 in neurons and T cells;3-HAA: increases/reduces ROS production, damages mitochondria; KYN: reduces ROS production, activates the SKN-1/Nrf2 pathway; KYNA: activates AHRs, activates GPR35, activates α7 nAChR. antagonizes NMDAR, antagonizes AMPAR, antagonizes rainate receptor, reduces ROS production; AA: increases/reduces ROS production, activates GPR109; XA: activates mGlu2/3R in neurons, activates GPCR; PA: reduces ROS production, promotes hippocampal NSC proliferation, differentiation, and neuronal maturation; IDO: bridges the KP and neuroinflammation

The KP also has four branches (see Fig. 1). In astrocytes, four KATs facilitate the irreversible conversion of KYN to KYNA [29]. In the neural networks of mammals, despite an abundance of numerous competitive amino acids, KAT II shows remarkable substrate selectivity for KYN and has optimal enzymatic activity under physiological pH conditions, thus being regarded as the main enzyme for KYNA synthesis [30]. KYNA is a key neuroactive metabolite in the KP, and its biological functions in the CNS have received extensive attention (see Fig. 2). Systemic subcutaneous administration of four classic convulsant agents—pentylenetetrazol (PTZ), pilocarpine, lycorine, and kainic acid—consistently elevated KYNA levels in vivo [31]. The endogenous concentration of KYNA in the brain typically ranges from nanomolar to low micromolar levels [32]. Even at nanomolar concentrations, KYNA can exert neuroprotective effects against excitotoxicity by antagonizing the glycine binding site on NMDA receptors, thereby reducing the brain's glutamate level by approximately 30–40% [33]. Therefore, the elevation of KYNA is likely to be a negative feedback regulation against epilepsy. However, antagonism at the glycine binding site can impair long-term potentiation and reduce persistent discharge activity in the prefrontal cortex, thereby damaging advanced cognitive functions such as working memory, learning, and information integration [34].

In microglia, KYNU facilitates the transformation of KYN into anthranilic acid (AA), which can generate 3HAA via non-specific hydroxylation [36]. AA is an endogenous agonist of GPR109A and can maintain myelin integrity by activating the AA-GPR109A-cPLA2 signaling axis [37, 38]. This branch also leads to the formation of cinnabarinic acid (CA), a neuroactive compound generated by the enzymatic oxidative polymerization of 3-HAA, a process often facilitated by peroxidases. CA functions as an orthosteric agonist of the mGlu4 receptor, binding to the glutamate-binding pocket within the N-terminal domain of the receptor and inhibiting cAMP production, thereby alleviating neuronal death caused by excitotoxicity [39].

KAT also catalyze the methylation of 3-HK to form xanthurenic acid (XA). XA exhibits significant antioxidant properties. A decline in XA levels suggests a diminished cellular antioxidant defense capacity [40]. XA activates group II metabotropic glutamate receptors (mGlu2 and mGlu3), inhibits cAMP production, and thereby negatively regulates neurotransmitter release [39]. Additionally, XA inhibits the vesicular glutamate transporter, preventing glutamate from entering synaptic vesicles and thereby reducing the release of synaptic glutamate [39].

3-HAA can also be converted to 2-amino-3-muconic acid-6-semialdehyde (AMS) by ACMSD, which is then transformed into picolinic acid (PIC) through a non-enzymatic reaction. PIC, as a neuroprotective metabolite, can significantly improve cognitive dysfunction induced by β₂-microglobulin and promote the proliferation of hippocampal neural stem/progenitor cells, guide their differentiation into the neuronal lineage, and support the maturation process of adult-born neurons [41].

## Roles of the KP in epilepsy

### The KP and neuroinflammation

**Fig. 3:**
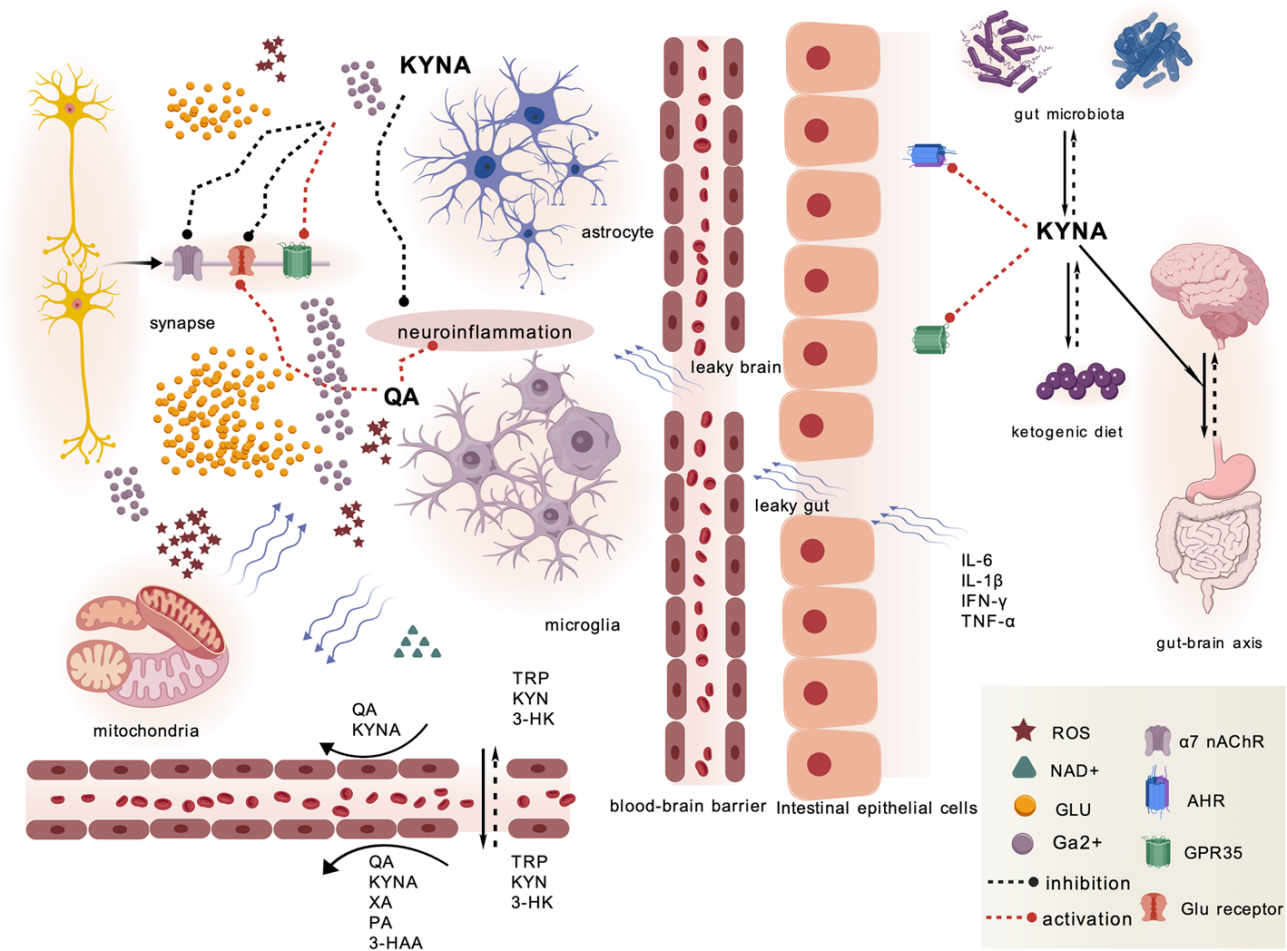
The primary target sites for two key metabolites of the KP (Created with BioGDP.com [35]) KYNA and QA are respectively the major metabolites in KP. Unlike TRP, KYN, and 3-HK, KYNA and QA exhibit limited permeability across the BBB, indicating that brain-derived KYNA and QA are primarily generated through the KP within the CNS. Within the brain, KYNA synthesis predominantly occurs in astrocytes, while QA is primarily produced in microglia. The effects of KYNA and QA exhibit mutual antagonism. KYNA can inhibit NMDA receptors and α7 nAChRs, activate GPR35, thereby reducing the generation of (ROS, intracellular Ca2 + levels, inflammatory responses, and suppressing glutamatergic neurotransmission. QA increases the generation of ROS, elevates intracellular Ca^2^^+^ levels, enhances inflammatory responses, and promotes glutamate secretion primarily through the activation of NMDA receptors and other mechanisms. The imbalance of QA levels affects the generation of NAD + downstream, leading to further aggravation of mitochondrial dysfunction and oxidative stress. In addition, KYNA can activate the AhR in the intestine and interact with intestinal microorganisms, possibly participating in the regulation of the central nervous system by ketogenic diets, becoming a part of the gut-brain axis

The IDO1 enzyme, which contains heme prosthetic groups, operates as a dioxygenase. It facilitates the oxidative breakage of the pyrrole ring in TRP via the oxidation of iron-bound molecular oxygen [42]. This process catalyzes the first rate-limiting step in the KP. A positive feedback loop exists between IDO and inflammation. Under conditions of neuroinflammation, pro-inflammatory cytokines (such as TNF-α, IFN-γ, and IL-6) induce excessive microglial activation, upregulate IDO1 expression, and promote increased QA synthesis [43]. Concurrently, astrocyte dysfunction leads to reduced synthesis of KYNA. Inflammatory factors such as IL-1β, IL-6, IL-8 and TNF-α are also positively correlated with the activity of KMO, further indicating that under this condition, KP metabolism tends to the branch of QA synthesis [44]. QA triggers a pro-inflammatory response in astrocytes (see Fig. 3), leading to the production of substantial amounts of monocyte chemoattractant protein-1, modest levels of regulated upon activation, normal T cell expressed and secreted, and IL-8, thereby promoting neuroinflammation [45]. Both KYNA and SZR104 can effectively inhibit the phagocytic activity of microglia induced by lipopolysaccharide (LPS). The KYNA analogue SZR104 can suppress the reactivity of microglia after status epilepticus [46].

In the serum of patients with idiopathic generalized epilepsy (such as juvenile myoclonic epilepsy), IDO activity remains elevated even in the absence of recent clinical seizures, suggesting that epilepsy may trigger an adaptive metabolic response [47]. KYN, produced by IDO, upregulates the expression of the enzyme itself by activating the AhR. This process involves interactions with signaling pathways such as NF-κB, thereby establishing a self-sustaining positive feedback loop [48]. The continuous activation of the aryl hydrocarbon receptor drives the differentiation of regulatory T cells, which helps to suppress excessive inflammatory responses [49, 50].

The mechanism driven by pro-inflammatory factors that leads to IDO1-induced QA/KYNA imbalance is prevalent in both acute and chronic inflammatory processes. After LPS-induced acute inflammation in the whole body or central nervous system, increased expression and activity of IDO1 induced by pro-inflammatory factors such as TNF-α, IL-6, IL-1β, and IFN-γ can be observed, accompanied by increased oxidative stress and decreased BDNF levels [51, 52]. Reduced levels of BDNF promote inflammatory responses and lead to sustained activation of IDO1. These manifestations are also observed in chronic inflammatory conditions, and inhibiting M1 microglial polarization reduces both IDO1 activation and its downstream effects, suggesting a close link between this process and microglia-driven inflammation [53, 54].

It is noteworthy that IDO can function as a component of the host innate immune system to restrict pathogen proliferation in the CNS independently of the KP. Infection with Toxoplasma gondii tachyzoites leads to a significant 40% increase in IDO activity, and subsequent inhibition of IDO by 1-MT results in a 1.2-fold increase in parasite proliferation [55]. IDO can be specifically expressed in the macrophages and microglia of the perivascular spaces and subarachnoid spaces of the brain, thereby linking it directly to phagocytic and inflammatory regulatory functions. Inhibition of IDO reduces the volume of microglia and macrophages and weakens their phagocytic function, accompanied by an increase in the secretion of pro-inflammatory factors such as IL-1β [56].

Knockout of IDO1 can delay the occurrence of lithium-pilocarpine-induced status epilepticus and significantly reduce the frequency, duration and severity of spontaneous recurrent seizures in the chronic phase [57]. IDO deficiency diminishes the production of KYN, the key precursor for QA synthesis in the brain, consequently resulting in reduced QA generation. The reduction of QA alleviated the levels of neuroinflammation and oxidative stress in the brain [57]. Contrastingly, in the TMEV-induced viral encephalitis epilepsy model, IDO knockout exhibited an opposing effect, resulting in a higher incidence of epilepsy [58]. This suggests a model-dependent role of IDO. In viral encephalitis, IDO1 is activated by the virus prior to the onset of epileptic seizures and may participate in the early host defense response. However, global knockout of IDO2 had no effect on epileptic seizures in mice with viral encephalitis, whereas specific deletion of IDO2 in microglia significantly reduced the incidence of epilepsy [59]. This may be due to IDO2 primarily driving the pro-inflammatory phenotype of microglia. These conflicting outcomes indicate that IDO1’s influence on epileptogenesis is context-dependent, varying with model type and inflammatory milieu.

### The KP and oxidative stress

The brain is very vulnerable to ROS, mainly due to its limited antioxidant defense capacity and high dependence on oxygen and redox processes [60]. Additionally, the significant presence of polyunsaturated fatty acid chains in cell membranes further exacerbates the brain's sensitivity to oxidative stress. Compared with ddY mice (a general-purpose outbred mouse strain), the activity of 3HAO was significantly increased in the CNS of epilepsy-prone EL mice (a genetic model of temporal lobe epilepsy) [61]. Abnormally elevated 3HAO activity was also observed in DBA/2 mice, a strain prone to audiogenic seizures [62]. QA, a downstream metabolite synthesized by 3HAO, increases intracellular calcium ion levels by activating NMDA receptors, ultimately leading to ROS generation, mitochondrial dysfunction, and apoptosis/necrosis [7, 14, 45]. Intrahippocampal injection of QA in rats leads to a significant increase in ROS within 4 h, which returns to normal levels within 24 h [63]. The overall increase in antioxidant capacity observed within 8 h is likely to reflect a compensatory response, which helps to alleviate the subsequent accumulation of ROS [64]. The QA-Fe^2^⁺ complex catalyzes ROS generation via Fenton-like reactions, initiating lipid peroxidation and cell membrane instability, which further damaging the vitality of astrocytes and neurons [5, 65, 66]. In addition, QA can disrupt the balance between reduced glutathione and oxidized glutathione, leading to impairment of the antioxidant defense system [67].

To maintain the balance of the KP, KYNA effectively combats the oxidative stress and excitotoxicity induced by QA and LPS through multiple mechanisms, including restoring Nrf2 levels, enhancing antioxidant defense, and improving mitochondrial function [64, 68]. It is worth noting that KYNA can significantly enhance the activities of three antioxidant enzymes (SOD2, CAT and GPx1) in the hypothalamus and hippocampus of sheep, providing experimental evidence for a deeper understanding of its antioxidant mechanism in the CNS [69]. KYNA also reduce calcium ion mobilization through GPR35, thereby alleviating mitochondrial damage and reducing the production of mitochondrial ROS [70].

Under pathological conditions, the metabolism of the KP shifts towards the synthesis of QA, leading to the gradual accumulation of ROS in the CNS. These ROS can further promote the secretion of pro-inflammatory cytokines such as IL-1β, TNF-α and IL-6 [71]. In addition, ROS can also weaken GABAergic inhibitory neurotransmission and enhance glutamatergic excitatory transmission, thereby reducing synaptic stability and increasing susceptibility to epilepsy [72]. Recurrent epileptic seizures, in turn, further promote the generation of ROS, thus forming a positive feedback loop that continuously aggravates the condition. Thus, a metabolic shift favoring QA over KYNA amplifies oxidative stress, perpetuating seizure vulnerability.

### The KP and the glutamatergic system

The neuroprotective effect of KYNA against neuronal overexcitation mainly stems from its ability to effectively antagonize the function of NMDA receptors, thereby inhibiting glutamate-mediated excitotoxicity (see Fig. 3). Studies have shown that exogenous administration of KYNA can inhibit glutamatergic activity in hippocampal samples from patients with mesial temporal lobe epilepsy and hippocampal sclerosis (MTLE-HS) [73]. This finding is consistent with the results of animal specimens: KYNA can inhibit the excessive excitation induced by glutamate receptors in the anterior temporal lobe and hippocampus samples of rats with TLE, but it does not affect the glutamate level in the neocortex. This regional difference may be attributed to the fact that the epileptogenic zone of TLE mainly involves subcortical structures rather than cortical areas [74]. In addition, the distribution, composition and functional status of glutamate receptors (especially the NMDA receptor subtype) also show regional differences among different brain regions.

In addition, local injection of KYNA into the caudate nucleus of rats significantly reduced the release of glutamate. Similarly, after systemic administration of a kynurenine hydroxylase inhibitor to increase KYNA levels in the brain, a decrease in glutamate output was also observed [33]. However, this effect is not mediated by glutamate receptors but may be related to nicotinic acetylcholine receptors (nAChRs). KYNA also exerts its anti-glutamatergic effect by antagonizing the α7nAChRs. Studies have confirmed that α7nAChRs are expressed in glutamatergic axon terminals in the human neocortex, and the activation of these receptors promotes the release of glutamate [75]. In addition, KYNA can act as an agonist of GPR35. In astrocytes, the activation of GPR35 by KYNA can reduce cAMP levels and regulate calcium transients, thereby reducing excitatory synaptic transmission [76].

QA disrupts the homeostasis of the brain's glutamatergic system by inhibiting the uptake of glutamate by astrocytes and down-regulating the expression of glutamine synthetase [77]. QA can also lead to excessive phosphorylation of cytoskeletal proteins [78–80], including glial fibrillary acidic protein, neurofilaments, and Tau protein. This process disrupts microtubule-dependent axonal transport, simultaneously causing delayed structural development of astrocytes, thereby impairing synaptic transmission, affecting synaptic plasticity, and intensifying neuronal hyperexcitability, promoting the occurrence of chronic epilepsy [81–83]. In summary, these findings highlight the crucial role of the KP balance (particularly the KYNA/QA ratio) in regulating glutamatergic tone and neuronal excitability. Since KYNA cannot directly pass through the BBB, the potential of its analogues in the treatment of epilepsy still needs to be verified through further research.

### The KP and gut-brain axis

There is a close bidirectional interaction between the KP and the gut microbiota (GM) (see Fig. 3). In the intestines of germ-free mice, the conversion of TRP to KYN is suppressed. After the intestinal flora returns to normal, the metabolic activities of the KP will also recover accordingly [84]. For instance, supplementation with *Bifidobacterium* can increase the concentration of KYNA in the plasma of rats [85]. The metabolic products of intestinal symbiotic bacteria (such as butyrate) can inhibit the transcription of IDO-1 driven by IFN-γ/pSTAT1 by down-regulating the level of STAT1 protein. It can directly inhibit the transcription of the IDO-1 gene without relying on STAT1, mainly by suppressing the activity of histone deacetylases [86].

During intestinal inflammation, pro-inflammatory cytokines such as IL-6 induce the expression of IDO1. The resulting KYNA acts on AhR, initiating negative feedback regulation and thereby reducing the levels of IL-1β and TNF-α in the intestine [87]. Reduction of intestinal TNFα can improve the hyperexcitability of neurons in mice [88], which in turn lowers the seizure threshold and reduces the duration of spontaneous seizures after status epilepticus [89]. In addition, KYN can stimulate the expression of GPR15 by activating AhR, a process mainly mediated by the KYN-AhR-FoxP3 axis [90]. In intestinal regulatory T cells, activated AhR works in concert with the lineage-determining transcription factor FoxP3 to upregulate the expression of GPR15. It is worth noting that recent studies have shown that activating GPR15 can regulate intestinal homeostasis and inflammation by modulating the migration of immune cells [91]. Intestinal inflammation may lead to increased intestinal permeability, allowing intestinal microbial products such as peptidoglycan to enter the bloodstream [92]. The fragments of peptidoglycan (such as muramyl dipeptide) can cross the BBB, stimulate microglia, and induce the synthesis of pro-inflammatory cytokines such as TNF-α/IL-1 [92]. These inflammatory factors promote the release of matrix metalloproteinase-9, which in turn degrades the tight junction protein Claudin-5, ultimately disrupting the integrity of the BBB, and leading to the phenomenon of "leaky brain" [93]. The aggravated brain edema and neuronal damage eventually induce epileptic seizures [94].

GPR35 is an important and highly expressed receptor on vagal nerve endings involved in gut-brain signaling, functioning as a sensor for signals derived from microbial metabolites [95]. When KYNA activates GPR35, the signal is transmitted upward to the CNS through the vagus nerve of the intestine [96]. This upward signal will be further transmitted to brain regions including the hypothalamus, amygdala and locus coeruleus [97]. Ultimately, this pathway can alleviate epileptic activity and activate the cholinergic anti-inflammatory pathway [98]. Simultaneously, activating the KYNA-GPR35 axis can significantly enrich the *Lactobacillus genus* in the intestine, regulate tyrosine and TRP metabolism, and alleviate neuroinflammation and neuronal apoptosis through the gut-brain axis [99].

In conclusion, the GM regulates the metabolism of the KP, and the metabolites of the KP, in turn, regulate the intestinal immune and inflammatory status. These interactions ultimately converge through the BBB and vagal afferent pathways, jointly influencing neuroinflammation and neuronal excitability, thereby promoting the occurrence of epilepsy and disease progression. These research results provide support for our view that chlorogenic acid, as a dietary polyphenol, alleviates hippocampal neurodegeneration, neuroinflammation and epileptic seizures in mice by increasing the production of short-chain fatty acids. In this study, changes in the GM were significantly correlated with the levels of multiple neurotransmitters, including KYN [100]. Clinical observations have found that in children with epilepsy, the ketogenic diet treatment increases the level of KYNA in the blood, and the degree of this increase is positively correlated with the clinical improvement of epilepsy symptoms [101].

## KP metabolites: a promising biomarker for epilepsy

Compared with the control group, the level of KYN in the cerebrospinal fluid (CSF) of epilepsy patients was significantly increased, while the concentrations of KYNA and TRP were significantly decreased (see Table 1) [99]. An elevated KYN/TRP ratio indicates enhanced IDO activity. In the brains of epilepsy patients, inflammatory factors activate IDO, leading to an imbalance within the KP, which is manifested as a decrease in the KYN/KYNA ratio. In this case, the antagonistic effect of KYNA on QA is insufficient, weakening the brain's defense against excitotoxicity. In the febrile subgroup, a higher KYN/TRP ratio reflects enhanced neuroinflammation [99]. In another study, the TRP-KYN pathway was one of the most significantly altered metabolic pathways in the CSF of patients with status epilepticus, with elevated levels of QA reflecting a more severe seizure state [100].The dysregulation of KP is not restricted to TLE. In CSF from patients with epileptic spasms, a decreased KYNA/KYN ratio is also observed, independent of brain structural abnormalities [102].Evidence from temporal lobe tissue specimens of patients with MTLE-HS further supports the view that the KP is imbalanced in patients with epilepsy [73].

**Table 1:** Alteration of KP metabolites and IDO among patients with different types of epileptic seizures

Group	The KP metabolites							The ratio		Sample	Refs.
	**IDO1**	**KYN**	**KYNA**	**QA**	**XA**	**AA**	**TRP**	**KYNA/KYN**	**KYN/TRP**		
Juvenile myoclonic epilepsy	**↑**	**-**					**↓**			Serum	[47]
Unclassified idiopathic generalized epilepsy	**↑**	**-**					**↓**			Serum	
Multifocal epilepsy	**-**	**-**					**↓**			Serum	
Extra -temporal lobe epilepsy	**-**	**-**					**↓**			Serum	
Temporal lobe epilepsy without hippocampal sclerosis	**↑**	**-**					**↓**			Serum	
Temporal lobe epilepsy with hippocampal sclerosis	**-**	**-**					**↓**			Serum	
Primary epilepsy	**↑**								**↑**	Serum, CSF	[57]
Epilepsy secondary to autoimmune encephalitis	**↑**								**↑**	Serum, CSF	
Status epilepticus	**↑**								**↑**	Serum, CSF	
Mesial temporal lobe epilepsy with hippocampal sclerosis		**-**	**↓**	**↑**			**↓**		**↑**	Hippocampus	[73]
Epileptic spasms (brain MRI results were categorized as follows: normal, focal cortical dysplasia FCD, brain atrophy, or other malformations and injuries)			**↓**					**↓**	**-**	CSF	[102]
Epileptic spasms patients who were steroid responders or partial responders			**↓**					**↓**		CSF	
Convulsive status epilepticus		**↑**	**↑**	**↑**						CSF	[103]
Refractory epilepsy responsive to ketogenic diet		**↓**	**↑**				**↓**	**↑**	-	Plasma	[101]
Developmental and epileptic encephalopathy, status epilepticus, generalised epilepsy, and focal epilepsy		**↑**	**↓**					**↓**	**↑**	CSF	[104]
Cerebral palsy with epilepsy			**↑**							Feces	[105]
Refractory epilepsy								**↓**		Plasma	[106]
Refractory epilepsy responsive to vagus nerve stimulation								**↑**		Plasma	
Infantile epileptic spasm syndrome (IESS) responsive to ACTH					**↑**					Plasma, feces	[107]
Intractable epilepsy responsive to vagus nerve stimulation						**↑**				Serum, CSF	[108]

KP metabolites can also be used for predicting therapeutic efficacy. Treatment with high-dose corticosteroids for 4 weeks usually relieves epileptic spasms, a predominant form of epileptic encephalopathy in infants. Compared with steroid-resistant patients, patients with epileptic spasms who respond to or partially respond to steroid treatment have a lower KYNA/KYN ratio in CSF [102]. Individuals exhibiting a more significant decrease in seizure frequency after adopting a ketogenic diet showed elevated serum KYNA levels, consistent with the anticonvulsant properties of KYNA [101]. Future investigations should systematically profile alterations of additional KP metabolites in paired CSF and serum samples from patients with epilepsy, as well as the sensitivity and specificity of KP metabolites as biomarkers to indicate specific types of epilepsy.

## The KP as the therapeutic target

### The IDO inhibition

1-methyl-tryptophan (1-MT) is a TRP analogue that can competitively inhibit the activity of IDO. Through the BDNF/TrkB pathway, I-MT promotes hippocampal neurogenesis and reduces the loss of dopaminergic neurons in the substantia nigra pars compacta [109]. Concurrently, I-MT promotes the activation of D2 like receptors, leading to enhanced GABAergic inhibition and suppressed glutamatergic excitability [109]. Neuronal excitability imbalance is a core pathophysiological mechanism of epileptic seizures. That is why we hypothesized that 1‑MT has therapeutic potential for mitigating epileptic seizures. The prediction subsequently validated by further study: 1-MT exerted multiple beneficial effects in TLE, including correcting the overactivation of BDNF/TrkB, reducing oxidative stress, restoring the activity of mitochondrial complex I/IV and reducing neuronal death, ultimately lowering the frequency and duration of epileptic seizures [19].

IDO is widely expressed in multiple organs, so systemic administration of IDO inhibitors may lead to extensive side effects. However, a preclinical safety study found that in IDO1 gene knockout mice, no genotype-related behavioral abnormalities, histopathological damage, or severe blood biochemical disorders were observed [110].

### The KMO inhibition

KMO is one of the key enzymes in KP, determining whether the metabolic flow leads to the production of QA or KYNA. During neuroinflammation, microRNAs such as miR-132/212 can negatively regulate the expression and activity of KMO, thereby limiting the excessive production of QA [7]. Diclofenac, as a non-selective COX inhibitor, also has the effect of inhibiting KMO activity. In the rotenone corneal kindling model, it reduces the level of QA by inhibiting KMO activity, thereby alleviating oxidative stress in the brain [111]. This is helpful in breaking the vicious cycle of neuroinflammation and oxidative stress driven by mitochondrial dysfunction in drug-resistant epilepsy [111]. The KMO inhibitor Ro 61–8048 is also capable of shifting the metabolic flux within the KP toward KYNA generation, thereby correcting the inherent metabolic imbalance [112]. While the frequency and severity of epileptic seizures were significantly reduced, the depressive behavior and cognitive level of mice were also improved [112].

Both chronic and acute inhibition of KMO do not affect overall energy metabolism, providing a safety rationale for the development of KMO inhibitors as therapeutic agents [113]. However, current findings in Drosophila demonstrate that KMO deficiency can directly lead to defects in mitochondrial morphology and function, independent of the KP [114]. In mammalian cells, KMO is involved in the post-translational regulation of DRP1, a key protein in mitochondrial fission [114]. Although the above phenomenon has not yet been confirmed in the human brain, future investigations should prioritize assessing the safety profile associated with KMO inhibition.

### KP and ASMs

Levetiracetam mainly regulates neurotransmitter release by binding to synaptic vesicle protein 2 A (SV2A). A study has found that it can also prevent the excitotoxicity, oxidative damage, abnormal neurotransmitter release and cell morphology damage caused by QA in the striatum of rats [115]. This suggests that modulation of the dysregulated KP metabolism may represent a potential mechanism underlying the antiepileptic effects of levetiracetam. Levetiracetam can enhance the production of KYNA and XA by astrocytes after IFN-γ intervention, while inhibiting the release of CA and QA [116]. KYNA and XA have anticonvulsant properties, while QA is a well-known proconvulsant metabolite. CA has dual and complex pharmacological properties, capable of exerting an anticonvulsant effect while also potentially triggering absence seizures [117, 118]. The inhibition of CA release partly explains the efficacy of levetiracetam in the treatment of absence seizures. Levetiracetam may exert an antidepressant effect indirectly by upregulating the production of KYNA and thereby modulating the function of the glutamatergic system. However, in fact, depression is one of its known side effects. Although the antagonistic effects on the NMDA receptors and the α7nAChRs are enhanced, the metabolic flow towards KP also leads to the depletion of 5-HT. Inhibiting IDO can improve levetiracetam-related depressive behavior, which will be discussed in detail below [119, 120].

Carbamazepine can selectively enhance the activity of KAT I in vitro, thereby promoting the synthesis of KYNA in brain cortex slices [121]. This mechanism may exert an auxiliary antiepileptic effect without relying on its function of blocking sodium channels in neurons. Phenobarbital, felbamate, phenytoin, and lamotrigine can also enhance the catalytic activity of the KAT I enzyme, thereby promoting the generation of KYNA in brain cortex slices, which indicates that KYNA may be involved in their antiepileptic effect [122].

However, analyses of brain tissue and blood samples from patients with drug-resistant epilepsy who underwent surgical resection revealed that carbamazepine has difficulty crossing the BBB and is metabolized by CYP3A4 enzymes within the BBB into QA (see Table 2) [123]. Therefore, the author infers that quinolinic acid, after entering the brain, would trigger drug resistance by activating neural excitatory pathways such as NMDA receptors [123]. This discovery provides new insights into the mechanism of drug resistance of ASMs. Targeting the KP to correct the unbalanced levels of its metabolites can reverse the reduced efficacy of multiple ASMs (such as levetiracetam, valproic acid, phenytoin, lamotrigine and carbamazepine) in epilepsy models associated with mitochondrial dysfunction [126].

**Table 2:** The mutual influence between ASMs and KPs

ASMs	The influence of drugs on KP	The influence of KP on drugs	Refs.
Levetiracetam	Increase the synthesis of KYNA and XA Inhibit the synthesis of CA and QA	Combining with IDO inhibitors alleviates their depressive side effects without compromising antiseizure efficacy	[116]
Carbamazepine	Enhance the activity of KAT and promote the synthesis of KYNA	It is metabolized into QA within the BBB and may further induce drug resistance by activating the excitatory pathway	[123]
Carbamazepine, phenytoin, phenobarbital, valproic acid, diazepam	Enhance the activity of KAT and promote the synthesis of KYNA	It can suppress the epileptic seizures induced by QA, but cannot prevent the neuronal death caused by QA. The combined use with neuroprotective agents (such as IDO/KMO inhibitors) can be considered	[121, 122, 124, 125]

In summary, regulating the KP balance may become a common mechanism for many existing ASMs and is expected to serve as a potential therapeutic strategy to overcome drug resistance. A variety of ASMs (such as carbamazepine, phenytoin, phenobarbital, valproic acid, flunarizine and diazepam) can suppress epileptic seizures induced by QA, but they cannot prevent the neuronal death caused by it [124, 125]. This indicates that treating such conditions requires a combined strategy that integrates ASMs with neuroprotective interventions. IDO inhibitors and KMO inhibitors can directly reduce the synthesis of QA, thereby achieving the above-mentioned purpose.

### The role of IDO in epilepsy comorbid with depression

The treatment of epilepsy combined with depression is rather complicated, as traditional antidepressants may trigger seizures, while certain ASMs (such as levetiracetam) have the side effect of causing depression [119]. Repeated intraperitoneal injection of subconvulsive doses of pentylenetetrazol (PTZ) in Swiss albino mice is an effective method for establishing a TLE model [127]. Recurrent epileptic seizures can activate proinflammatory cytokines, such as IL-1β, IL-6, and TNF-α, thereby promoting IDO upregulation and shifting TRP metabolism toward KP. This metabolic shift is characterized by a decreased 5-HT/TRP ratio and an increased KYN/TRP ratio, ultimately leading to depression-related behaviors [120]. Although sodium valproate significantly reduces the severity of epileptic seizures, monotherapy cannot alleviate the comorbid depressive symptoms in epilepsy patients [127]. Therefore, we propose that targeting IDO as a drug target may be a potentially effective and safe therapeutic approach.

1-MT, a synthetic inhibitor of IDO, can effectively reverse depressive behaviors comorbid with epilepsy without exacerbating epileptic seizures [128]. Antibiotics like minocycline can reduce inflammatory factors such as IL-1β and IL-6, indirectly inhibiting the activity of IDO, thereby restoring the ratio of 5-HT/KYN. Such drugs can be used in combination with sodium valproate as a treatment option for epilepsy comorbid with depression [128, 129]. Some plant-derived natural compounds, such as ferulic acid and quercetin, can alleviate levetiracetam-induced depressive symptoms without compromising its antiepileptic efficacy by inhibiting neuroinflammation-mediated activation of IDO [119, 120].

**Fig. 4:**
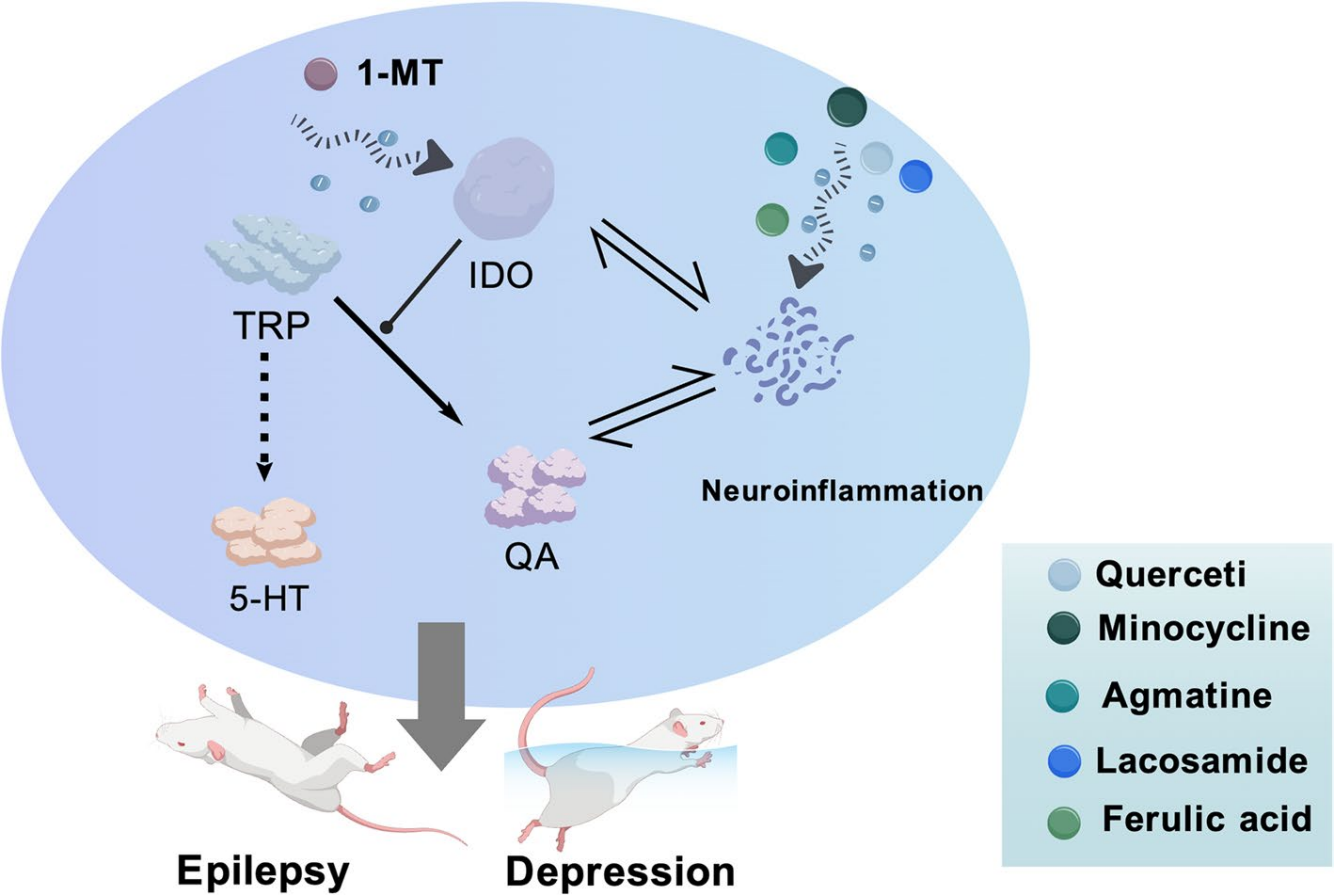
IDO is a potential therapeutic target for treating epilepsy comorbid with depression (Created with BioGDP.com [35]). Increased IDO activity shifts TRP metabolism towards the KP, resulting in reduced 5-HT production and elevated QA levels, which contributes to the development of epilepsy comorbid with depression. Furthermore, a vicious cycle between IDO activation and neuroinflammatory processes further exacerbates epilepsy comorbid with depression. The figure illustrates six pharmacological agents that inhibit IDO, thereby potentially alleviating epilepsy comorbid with depression

Agmatine can inhibit the overactivation of IDO, increase 5-HT levels, and reduce the abnormally elevated glutamate/GABA ratio, ultimately exerting anti-epileptic effects while also improving depressive and cognitive symptoms [130]. Lacosamide can selectively enhance the slow inactivation of sodium channels and is a widely used ASM in clinical practice. Nevertheless, in the model induced by pilocarpine and LPS, lacosamide can significantly reduce the expression levels of pro-inflammatory cytokines IL-1β and IL-6 in the hippocampus and decrease the accumulation of KP metabolites [131]. Therefore, targeting IDO in the KP represents a promising strategy for developing therapies with dual efficacy in the treatment of epilepsy and depression (Fig. 4).

## Conclusions

The KP is involved in the pathophysiological process of epilepsy by regulating neuroinflammation, glutamatergic neurotransmission, oxidative stress and the brain-gut axis. with QA being overproduced and KYNA insufficient to counteract its excitotoxic effects, the susceptibility to and severity of epileptic seizures will increase. Clinical evidence also indicates that alterations in KP metabolites can be found in both the serum and CSF of patients with epilepsy, and these changes are consistent with clinical symptoms. Theoretically, it is feasible to treat epilepsy by targeting the key enzymes in the KP, a fact that has been confirmed in animal studies. It should be particularly noted that the IDO-mediated shift of TRP metabolism towards the KP is a key mechanism linking epilepsy with comorbid depression. Multiple drugs have demonstrated therapeutic potential in animal models by inhibiting IDO activity, leading to concurrent improvements in epileptic seizures and depressive-like behaviors. Therefore, a deeper understanding of the role of the KP in the CNS, especially under epileptic conditions, offers a promising avenue for the development of novel ASMs.

## Limitations and future perspectives

However, the current evidence mainly comes from animal models, and there may be significant differences in the metabolism of the KP among different species. Therefore, translational medicine necessitates more robust clinical evidence to validate these findings in human populations. Furthermore, some research findings are still based on theoretical inferences. For instance, carbamazepine can be metabolized into QA at the BBB. Theoretically, it is speculated that the series of reactions triggered by QA once it enters the brain might be the molecular basis of epilepsy drug resistance, but this mechanism still awaits further research for verification. While KYNA antagonizes NMDA receptors to resist excitotoxicity, it can also affect cognitive function. Therefore, the role of KP in various neurological comorbidities such as epilepsy-related cognitive decline deserves further discussion. In addition, the dynamic changes of KP in different subtypes of epilepsy, different brain regions and disease stages are not yet fully clear. Inhibition of KMO does not affect the overall energy metabolism of fruit flies, but KMO is involved in the post-translational regulation of key proteins for mitochondrial fission in mammalian cells. IDO is expressed in multiple organs. Although no severe blood biochemical disorders related to IDO gene knockout were observed in mice, future studies should focus on the safety of these key enzyme inhibitors while exploring their efficacy in humans. In the future, the different ratios of KP metabolites in CSF or blood can be used as markers for the analysis of epilepsy and the prediction of therapeutic effects, promoting individualized treatment.

## Data Availability

No datasets were generated or analysed during the current study.

## Abbreviations

AA: Anthranilic acid
ACMS: α-amino-α-carboxymuconic-ω-semialdehyde
AhR: Aryl hydrocarbon receptor
AMS: 2-amino-3-muconic acid-6-semialdehyde
ASMs: Antiseizure medications
BBB: Blood-brain barriers
CA: Cinnabarinic acid
CNS: Central nervous system reactions
CSF: Cerebrospinal fluid
GM: Gut microbiota
GPR35: G protein-coupled receptor 35
IDO: Indoleamine 2,3-dioxygenase
KAT: Kynurenine aminotransferase
KMO: Kynurenine monooxygenase
KP: Kynurenine pathway
KYN: Kynurenine
KYNA: Kynurenic acid
KYNU: Kynureninase
LPS: Lipopolysaccharide
mGlu: Metabotropic glutamate
MTLE-HS: Mesial temporal lobe epilepsy and hippocampal sclerosis
nAChRs: Nicotinic acetylcholine receptors
NMDA: N-Methyl-D-aspartate
PIC: Picolinic acid
PTZ: Pentylenetetrazol
QA: Quinolinic acid
ROS: Reactive oxygen species
SV2A: Synaptic vesicle protein 2A
TDO: Tryptophan 2,3-dioxygenase
TRP: Tryptophan
XA: Xanthurenic acid
1-MT: 1-methyl-tryptophan
3-HAO: 3-hydroxyanthranilic acid 3,4-dioxygenase
3-HK: 3-hydroxykynurenine
3HAA: 3-hydroxyanthranilic acid
5-HT: Serotonin

## References

[1] Leonardi M, Martelletti P, Burstein R, Fornari A, Grazzi L, Guekht A, et al. The World Health Organization intersectoral global action plan on epilepsy and other neurological disorders and the headache revolution: from headache burden to a global action plan for headache disorders. J Headache Pain. 2024;25:4. 38178049 10.1186/s10194-023-01700-3 PMC10768290

[2] Rashid H, Upadhyay AD, Pandey RM, Katyal J. Point prevalence of depression in persons with active epilepsy and impact of methodological moderators: a systematic review and meta-analysis. Epilepsy Behav. 2021;125:108394. 34794012 10.1016/j.yebeh.2021.108394

[3] Marson A, Burnside G, Appleton R, Smith D, Leach JP, Sills G, et al. The SANAD II study of the effectiveness and cost-effectiveness of levetiracetam, zonisamide, or lamotrigine for newly diagnosed focal epilepsy: an open-label, non-inferiority, multicentre, phase 4, randomised controlled trial. Lancet. 2021;397:1363–74. 33838757 10.1016/S0140-6736(21)00247-6 PMC8047799

[4] Tavares RG, Schmidt AP, Abud J, Tasca CI, Souza DO. In vivo quinolinic acid increases synaptosomal glutamate release in rats: reversal by guanosine. Neurochem Res. 2005;30:439–44. 16076013 10.1007/s11064-005-2678-0

[5] St’astný F, Lisý V, Mares V, Lisá V, Balcar VJ, Santamaría A. Quinolinic acid induces NMDA receptor-mediated lipid peroxidation in rat brain microvessels. Redox Rep Commun Free Radical Res. 2004;9:229–33. 10.1179/135100004225006001 15479567

[6] Bettini E, De Martin S, Mattarei A, Pappagallo M, Stahl SM, Bifari F, et al. The N-methyl-D-aspartate receptor blocker REL-1017 (esmethadone) reduces calcium influx induced by glutamate, quinolinic acid, and gentamicin. Pharmaceuticals (Basel). 2022;15:882. 35890179 10.3390/ph15070882 PMC9319291

[7] Kezai AM, Badiane PY, Hennart B, Allorge D, Marion S, Hébert SS. MicroRNA-132 regulates quinolinic acid production in the brain during LPS-induced neuroinflammation. Front Immunol. 2025;16:1644783. 40934002 10.3389/fimmu.2025.1644783 PMC12417171

[8] Goel F, Dobhal V, Singh S. Protective effects of diacerein against quinolinic acid-induced Huntington’s disease-like symptoms in adult zebrafish by targeting GSK-3β signalling. Naunyn Schmiedebergs Arch Pharmacol. 2025;398:10799–821. 40042555 10.1007/s00210-025-03976-5

[9] Lo Y-C, Lin C-L, Fang W-Y, Lőrinczi B, Szatmári I, Chang W-H, et al. Effective activation by kynurenic acid and its aminoalkylated derivatives on M-type K+ current. Int J Mol Sci. 2021;22:1300. 33525680 10.3390/ijms22031300 PMC7865226

[10] Wu C, Diao M, Yu S, Xi S, Zheng Z, Cao Y, et al. Gut microbial tryptophan metabolism is involved in post-cardiac arrest brain injury via pyroptosis modulation. CNS Neurosci Ther. 2025;31:e70381. 40260682 10.1111/cns.70381 PMC12012640

[11] Dhureja M, Arthur R, Soni D, Upadhayay S, Temgire P, Kumar P. Calcium channelopathies in neurodegenerative disorder: an untold story of RyR and SERCA. Expert Opin Ther Targets. 2023;27:1159–72. 37971192 10.1080/14728222.2023.2277863

[12] Yang W, Cui H, Wang C, Wang X, Yan C, Cheng W. A review of the pathogenesis of epilepsy based on the microbiota-gut-brain-axis theory. Front Mol Neurosci. 2024;17:1454780. 39421261 10.3389/fnmol.2024.1454780 PMC11484502

[13] Sarangdhar M, Akel S, Hosseini Ashtiani S, Axelsson M, Zelano J. Association between pro-inflammatory proteins and neurofilament in plasma from persons with epilepsy. BMC Med. 2025;23:554. 41083978 10.1186/s12916-025-04425-z PMC12519617

[14] Tassan Mazzocco M, Murtaj V, Martins D, Schellino R, Coliva A, Toninelli E, et al. Exploring the neuroprotective effects of montelukast on brain inflammation and metabolism in a rat model of quinolinic acid-induced striatal neurotoxicity. J Neuroinflammation. 2023;20:34. 36782185 10.1186/s12974-023-02714-z PMC9923670

[15] Ferreira FS, Dos Santos TM, Ramires Junior OV, Silveira JS, Schmitz F, Wyse ATS. Quinolinic acid impairs redox homeostasis, bioenergetic, and cell signaling in rat striatum slices: prevention by coenzyme Q10. Neurotox Res. 2022;40:473–84. 35239160 10.1007/s12640-022-00484-9

[16] Cao Y, Zhao K, Zhao Y, Ni Y, Zhang Z, Yuan X, et al. Arbutin ameliorated depression by inhibiting neuroinflammation and modulating intestinal flora. Phytomedicine. 2025;145:156944. 40554291 10.1016/j.phymed.2025.156944

[17] Santos JMD, Touguinha L, Bridi R, Andreazza AC, Bick DLU, Davidson CB, et al. Could the inhibition of systemic NLRP3 inflammasome mediate central redox effects of yerba mate? An in silico and pre-clinical translational approach. J Ethnopharmacol. 2025;344:119518. 39987999 10.1016/j.jep.2025.119518

[18] Miziak B, Czuczwar SJ, Pluta R. Comorbid epilepsy and depression-pharmacokinetic and pharmacodynamic drug interactions. Front Pharmacol. 2022;13:988716. 36278185 10.3389/fphar.2022.988716 PMC9585163

[19] Xu J, Wei L, Fu J, Kong Z, Cai L. IDO activation affects BDNF/TrkB signaling pathway, oxidative stress, and mitochondrial enzymatic activities in temporal lobe epilepsy. Curr Issues Mol Biol. 2025;47:764. 41020886 10.3390/cimb47090764 PMC12468408

[20] Cortés Malagón EM, López Ornelas A, Olvera Gómez I, Bonilla Delgado J. The kynurenine pathway, aryl hydrocarbon receptor, and Alzheimer’s disease. Brain Sci. 2024;14:950. 39335444 10.3390/brainsci14090950 PMC11429728

[21] Savitz J. The kynurenine pathway: a finger in every pie. Mol Psychiatry. 2020;25:131–47. 30980044 10.1038/s41380-019-0414-4 PMC6790159

[22] Ball HJ, Jusof FF, Bakmiwewa SM, Hunt NH, Yuasa HJ. Tryptophan-catabolizing enzymes - party of three. Front Immunol. 2014;5:485. 25346733 10.3389/fimmu.2014.00485 PMC4191572

[23] Kim SS, Kim S, Kim Y, Ha Y, Lee H, Im H, et al. Neurotoxic effects of citronellol induced by the conversion of kynurenine to 3-hydroxykynurenine. J Hazard Mater. 2025;486:136965. 39733753 10.1016/j.jhazmat.2024.136965

[24] Colín-González AL, Maldonado PD, Santamaría A. 3-hydroxykynurenine: an intriguing molecule exerting dual actions in the central nervous system. Neurotoxicology. 2013;34:189–204. 23219925 10.1016/j.neuro.2012.11.007

[25] Reyes-Ocampo J, Ramírez-Ortega D, Cervantes GIV, Pineda B, Balderas PMdeO, González-Esquivel D, et al. Mitochondrial dysfunction related to cell damage induced by 3-hydroxykynurenine and 3-hydroxyanthranilic acid: non-dependent-effect of early reactive oxygen species production. Neurotoxicology. 2015;50:81–91. 26254737 10.1016/j.neuro.2015.08.003

[26] Arbatova J, D’Amato E, Vaarmann A, Zharkovsky A, Reeben M. Reduced serotonin and 3-hydroxyanthranilic acid levels in serum of cystatin B-deficient mice, a model system for progressive myoclonus epilepsy. Epilepsia. 2005;46(Suppl 5):49–51. 15987253 10.1111/j.1528-1167.2005.01008.x

[27] Palzer L, Bader JJ, Angel F, Witzel M, Blaser S, McNeil A, et al. Alpha-amino-beta-carboxy-muconate-semialdehyde decarboxylase controls dietary niacin requirements for NAD+ synthesis. Cell Rep. 2018;25:1359-1370.e4. 30380424 10.1016/j.celrep.2018.09.091 PMC9805792

[28] Schwarcz R, Brush GS, Foster AC, French ED. Seizure activity and lesions after intrahippocampal quinolinic acid injection. Exp Neurol. 1984;84:1–17. 6705878 10.1016/0014-4886(84)90001-3

[29] Han Q, Cai T, Tagle DA, Li J. Structure, expression, and function of kynurenine aminotransferases in human and rodent brains. Cell Mol Life Sci. 2010;67:353–68. 19826765 10.1007/s00018-009-0166-4 PMC2867614

[30] Guidetti P, Amori L, Sapko MT, Okuno E, Schwarcz R. Mitochondrial aspartate aminotransferase: a third kynurenate-producing enzyme in the mammalian brain. J Neurochem. 2007;102:103–11. 17442055 10.1111/j.1471-4159.2007.04556.x

[31] Wu HQ, Schwarcz R. Seizure activity causes elevation of endogenous extracellular kynurenic acid in the rat brain. Brain Res Bull. 1996;39:155–62. 8866691 10.1016/0361-9230(95)02087-x

[32] Moroni F, Russi P, Lombardi G, Beni M, Carlà V. Presence of kynurenic acid in the mammalian brain. J Neurochem. 1988;51:177–80. 3379401 10.1111/j.1471-4159.1988.tb04852.x

[33] Carpenedo R, Pittaluga A, Cozzi A, Attucci S, Galli A, Raiteri M, et al. Presynaptic kynurenate-sensitive receptors inhibit glutamate release. Eur J Neurosci. 2001;13:2141–7. 11422455 10.1046/j.0953-816x.2001.01592.x

[34] Phenis D, Vunck SA, Valentini V, Arias H, Schwarcz R, Bruno JP. Activation of alpha7 nicotinic and NMDA receptors is necessary for performance in a working memory task. Psychopharmacology. 2020;237:1723–35. 32162104 10.1007/s00213-020-05495-y PMC7313359

[35] Jiang S, Li H, Zhang L, Mu W, Zhang Y, Chen T, et al. Generic diagramming platform (GDP): a comprehensive database of high-quality biomedical graphics. Nucleic Acids Res. 2025;53:D1670–6. 39470721 10.1093/nar/gkae973 PMC11701665

[36] Ogyu K, Kubo K, Noda Y, Iwata Y, Tsugawa S, Omura Y, et al. Kynurenine pathway in depression: a systematic review and meta-analysis. Neurosci Biobehav Rev. 2018;90:16–25. 29608993 10.1016/j.neubiorev.2018.03.023

[37] Oxenkrug G, Forester B. Anthranilic acid, a GPR109A agonist, and schizophrenia. Int J Tryptophan Res. 2024;17:11786469241239125. 38532858 10.1177/11786469241239125 PMC10964450

[38] Oxenkrug G. Anthranilic acid-G-protein coupled receptor109A-cytosolic phospholipase A2-myelin-cognition cascade: a new target for the treatment/prevention of cognitive impairment in schizophrenia, dementia, and aging. Int J Mol Sci. 2024;25:13269. 39769034 10.3390/ijms252413269 PMC11675959

[39] Fazio F, Lionetto L, Curto M, Iacovelli L, Copeland CS, Neale SA, et al. Cinnabarinic acid and xanthurenic acid: two kynurenine metabolites that interact with metabotropic glutamate receptors. Neuropharmacology. 2017;112:365–72. 27342123 10.1016/j.neuropharm.2016.06.020

[40] Kubicova L, Hadacek F, Bachmann G, Weckwerth W, Chobot V. Coordination complex formation and redox properties of kynurenic and xanthurenic acid can affect brain tissue homeodynamics. Antioxidants. 2019;8:476. 31614581 10.3390/antiox8100476 PMC6826357

[41] Zhou G-J, Tang Y-Y, Zuo J-X, Yi T, Tang J-P, Zhang P, et al. Itaconate alleviates β2-microglobulin-induced cognitive impairment by enhancing the hippocampal amino-β-carboxymuconate-semialdehyde-decarboxylase/picolinic acid pathway. Biochem Pharmacol. 2022;202:115137. 35700758 10.1016/j.bcp.2022.115137

[42] Sugimoto H, Oda S, Otsuki T, Hino T, Yoshida T, Shiro Y. Crystal structure of human indoleamine 2,3-dioxygenase: catalytic mechanism of O_2_ incorporation by a heme-containing dioxygenase. Proc Natl Acad Sci U S A. 2006;103:2611–6. 16477023 10.1073/pnas.0508996103 PMC1413787

[43] Sodhi RK, Bansal Y, Singh R, Saroj P, Bhandari R, Kumar B, et al. IDO-1 inhibition protects against neuroinflammation, oxidative stress and mitochondrial dysfunction in 6-OHDA induced murine model of Parkinson’s disease. Neurotoxicology. 2021;84:184–97. 33774066 10.1016/j.neuro.2021.03.009

[44] Yang S, Han J, Ye Z, Zhou H, Yan Y, Han D, et al. The correlation of inflammation, tryptophan-kynurenine pathway, and suicide risk in adolescent depression. Eur Child Adolesc Psychiatry. 2025;34:1557–67. 39287643 10.1007/s00787-024-02579-4

[45] Guillemin GJ, Croitoru-Lamoury J, Dormont D, Armati PJ, Brew BJ. Quinolinic acid upregulates chemokine production and chemokine receptor expression in astrocytes. Glia. 2003;41:371–81. 12555204 10.1002/glia.10175

[46] Lajkó N, Kata D, Szabó M, Mátyás A, Dulka K, Földesi I, et al. Sensitivity of rodent microglia to kynurenines in models of epilepsy and inflammation in vivo and in vitro: microglia activation is inhibited by kynurenic acid and the synthetic analogue SZR104. Int J Mol Sci. 2020;21:9333. 33297593 10.3390/ijms21239333 PMC7731372

[47] Liimatainen S, Lehtimäki K, Raitala A, Peltola M, Oja SS, Peltola J, et al. Increased indoleamine 2,3-dioxygenase (IDO) activity in idiopathic generalized epilepsy. Epilepsy Res. 2011;94:206–12. 21377330 10.1016/j.eplepsyres.2011.02.003

[48] Vogel CFA, Goth SR, Dong B, Pessah IN, Matsumura F. Aryl hydrocarbon receptor signaling mediates expression of indoleamine 2,3-dioxygenase. Biochem Biophys Res Commun. 2008;375:331–5. 18694728 10.1016/j.bbrc.2008.07.156 PMC2583959

[49] Vogel CFA, Wu D, Goth SR, Baek J, Lollies A, Domhardt R, et al. Aryl hydrocarbon receptor signaling regulates NF-κB RelB activation during dendritic-cell differentiation. Immunol Cell Biol. 2013;91:568–75. 23999131 10.1038/icb.2013.43 PMC3806313

[50] Mezrich JD, Fechner JH, Zhang X, Johnson BP, Burlingham WJ, Bradfield CA. An interaction between kynurenine and the aryl hydrocarbon receptor can generate regulatory T cells. J Immunol. 2010;185:3190–8. 20720200 10.4049/jimmunol.0903670 PMC2952546

[51] Mallik SB, Mudgal J, Kinra M, Hall S, Grant GD, Anoopkumar-Dukie S, et al. Involvement of indoleamine 2, 3-dioxygenase (IDO) and brain-derived neurotrophic factor (BDNF) in the neuroprotective mechanisms of ferulic acid against depressive-like behaviour. Metab Brain Dis. 2023;38:2243–54. 37490224 10.1007/s11011-023-01267-7 PMC10504153

[52] Dobos N, de Vries EFJ, Kema IP, Patas K, Prins M, Nijholt IM, et al. The role of indoleamine 2,3-dioxygenase in a mouse model of neuroinflammation-induced depression. J Alzheimers Dis. 2012;28:905–15. 22112548 10.3233/JAD-2011-111097

[53] de Paiva IHR, Maciel LM, da Silva RS, Mendonça IP, de Souza JRB, Peixoto CA. Prebiotics modulate the microbiota-gut-brain axis and ameliorate anxiety and depression-like behavior in HFD-fed mice. Food Res Int. 2024;182:114153. 38519181 10.1016/j.foodres.2024.114153

[54] Lu R, Zhang L, Wang H, Li M, Feng W, Zheng X. Echinacoside exerts antidepressant-like effects through enhancing BDNF-CREB pathway and inhibiting neuroinflammation via regulating microglia M1/M2 polarization and JAK1/STAT3 pathway. Front Pharmacol. 2022;13:993483. 36686689 10.3389/fphar.2022.993483 PMC9846169

[55] Jesus LB, Santos AB, Jesus EEV, Santos RGD, Grangeiro MS, Bispo-da-Silva A, et al. IDO, COX and iNOS have an important role in the proliferation of *Neospora caninum* in neuron/glia co-cultures. Vet Parasitol. 2019;266:96–102. 30736955 10.1016/j.vetpar.2019.01.003

[56] Ji R, Ma L, Chen X, Sun R, Zhang L, Saiyin H, et al. Characterizing the distributions of IDO-1 expressing macrophages/microglia in human and murine brains and evaluating the immunological and physiological roles of IDO-1 in RAW264.7/BV-2 cells. PLoS One. 2021;16:e0258204. 34735466 10.1371/journal.pone.0258204 PMC8568167

[57] Deng N, Hu J, Hong Y, Ding Y, Xiong Y, Wu Z, et al. Indoleamine-2,3-Dioxygenase 1 Deficiency Suppresses Seizures in Epilepsy. Front Cell Neurosci Front Cell Neurosci. 2021;15:638854. 10.3389/fncel.2021.638854 PMC7935521 33679331

[58] Juda MB, Brooks AK, Towers AE, Freund GG, McCusker RH, Steelman AJ. Indoleamine 2,3-dioxygenase 1 deletion promotes Theiler’s virus-induced seizures in C57BL/6J mice. Epilepsia. 2019;60:626–35. 30770561 10.1111/epi.14675 PMC8273875

[59] MacDowell Kaswan ZA, Brooks AK, Hurtado M, Chen EY, Steelman AJ, McCusker RH. Microglia-specific Ido2 deficiency attenuates ictogenesis in the TMEV model of viral encephalitis. Brain Behav Immun. 2025;129:839–56. 40712946 10.1016/j.bbi.2025.07.017 PMC13310017

[60] Gupta R, Soni D, Upadhayay S, Dhureja M, Kumar P. Impact of noscapine on halting the progression of pentylenetetrazole induced kindling epilepsy in mice. Clin Exp Pharmacol Physiol. 2023;50:984–91. 37724453 10.1111/1440-1681.13825

[61] Nakano K, Takahashi S, Mizobuchi M, Kuroda T, Masuda K, Kitoh J. High levels of quinolinic acid in brain of epilepsy-prone E1 mice. Brain Res. 1993;619:195–8. 8374778 10.1016/0006-8993(93)91612-v

[62] Eastman CL, Urbańska EM, Chapman AG, Schwarcz R. Differential expression of the astrocytic enzymes 3-hydroxyanthranilic acid oxygenase, kynurenine aminotransferase and glutamine synthetase in seizure-prone and non-epileptic mice. Epilepsy Res. 1994;18:185–94. 7805640 10.1016/0920-1211(94)90039-6

[63] Ganzella M, Jardim FM, Boeck CR, Vendite D. Time course of oxidative events in the hippocampus following intracerebroventricular infusion of quinolinic acid in mice. Neurosci Res. 2006;55:397–402. 16766071 10.1016/j.neures.2006.05.003

[64] Ferreira FS, Biasibetti-Brendler H, Pierozan P, Schmitz F, Bertó CG, Prezzi CA, et al. Kynurenic acid restores Nrf2 levels and prevents quinolinic acid-induced toxicity in rat striatal slices. Mol Neurobiol. 2018;55:8538–49. 29564809 10.1007/s12035-018-1003-2

[65] Heron P, Daya S. 17Beta-estradiol protects against quinolinic acid-induced lipid peroxidation in the rat brain. Metab Brain Dis. 2000;15:267–74. 11383551 10.1023/a:1011119107765

[66] Yan E, Castillo-Meléndez M, Smythe G, Walker D. Quinolinic acid promotes albumin deposition in Purkinje cell, astrocytic activation and lipid peroxidation in fetal brain. Neuroscience. 2005;134:867–75. 16026935 10.1016/j.neuroscience.2005.04.056

[67] Rodríguez-Martínez E, Camacho A, Maldonado PD, Pedraza-Chaverrí J, Santamaría D, Galván-Arzate S, et al. Effect of quinolinic acid on endogenous antioxidants in rat corpus striatum. Brain Res. 2000;858:436–9. 10708698 10.1016/s0006-8993(99)02474-9

[68] Gao Y, Guo X, Zhou Y, Du J, Lu C, Zhang L, et al. Kynurenic acid inhibits macrophage pyroptosis by suppressing ROS production via activation of the NRF2 pathway. Mol Med Rep. 2023;28:211. 37772394 10.3892/mmr.2023.13098 PMC10552067

[69] Misztal T, Roszkowicz-Ostrowska K, Kowalczyk P, Młotkowska P, Marciniak E. Kynurenic acid modulates the expression of genes and the activity of cellular antioxidant enzymes in the hypothalamus and hippocampus in sheep. Int J Mol Sci. 2024;25:9428. 39273374 10.3390/ijms25179428 PMC11395064

[70] Sun T, Xie R, He H, Xie Q, Zhao X, Kang G, et al. Kynurenic acid ameliorates NLRP3 inflammasome activation by blocking calcium mobilization via GPR35. Front Immunol. 2022;13:1019365. 36311752 10.3389/fimmu.2022.1019365 PMC9606686

[71] Parsons ALM, Bucknor EMV, Castroflorio E, Soares TR, Oliver PL, Rial D. The interconnected mechanisms of oxidative stress and neuroinflammation in epilepsy. Antioxidants. 2022;11:157. 35052661 10.3390/antiox11010157 PMC8772850

[72] Fabisiak T, Patel M. Crosstalk between neuroinflammation and oxidative stress in epilepsy. Front Cell Dev Biol. 2022;10:976953. 36035987 10.3389/fcell.2022.976953 PMC9399352

[73] Dey S, Banerjee Dixit A, Tripathi M, Doddamani RS, Sharma MC, Lalwani S, et al. Altered hippocampal kynurenine pathway metabolism contributes to hyperexcitability in human mesial temporal lobe epilepsy-hippocampal sclerosis. Br J Pharmacol. 2021;178:3959–76. 33990935 10.1111/bph.15534

[74] Dey S, Dubey V, Dixit AB, Tripathi M, Chandra PS, Banerjee J. Differential levels of tryptophan-kynurenine pathway metabolites in the hippocampus, anterior temporal lobe, and neocortex in an animal model of temporal lobe epilepsy. Cells. 2022;11:3560. 36428989 10.3390/cells11223560 PMC9688794

[75] Wang Y, Wu W, Zeng F, Meng X, Peng M, Wang J, et al. The role of kynurenine pathway metabolism mediated by exercise in the microbial-gut-brain axis in Alzheimer’s disease. Exp Neurol. 2025;384:115070. 39603488 10.1016/j.expneurol.2024.115070

[76] Berlinguer-Palmini R, Masi A, Narducci R, Cavone L, Maratea D, Cozzi A, et al. GPR35 activation reduces Ca2+ transients and contributes to the kynurenic acid-dependent reduction of synaptic activity at CA3-CA1 synapses. PLoS ONE. 2013;8:e82180. 24312407 10.1371/journal.pone.0082180 PMC3843712

[77] Ting KK, Brew BJ, Guillemin GJ. Effect of quinolinic acid on human astrocytes morphology and functions: implications in Alzheimer’s disease. J Neuroinflammation. 2009;6:36. 20003262 10.1186/1742-2094-6-36 PMC2797503

[78] Nicholls T, Lacey B, Nitsos I, Smythe G, Walker DW. Regional changes in kynurenic acid, quinolinic acid, and glial fibrillary acidic protein concentrations in the fetal sheep brain after experimentally induced placental insufficiency. Am J Obstet Gynecol. 2001;184:203–8. 11174503 10.1067/mob.2001.108862

[79] Esmaeili S, Ghobadi N, Akbari V, Moradi S, Shahlaie M, Ghobadi S, et al. Pyridine-2,3-dicarboxylate, quinolinic acid, induces 1N4R Tau amyloid aggregation in vitro: another evidence for the detrimental effect of the inescapable endogenous neurotoxin. Chem Biol Interact. 2020;315:108884. 31678113 10.1016/j.cbi.2019.108884

[80] Joisten N, Rademacher A, Warnke C, Proschinger S, Schenk A, Walzik D, et al. Exercise diminishes plasma neurofilament light chain and reroutes the kynurenine pathway in multiple sclerosis. Neurol Neuroimmunol Neuroinflamm. 2021;8:e982. 33782190 10.1212/NXI.0000000000000982 PMC8054957

[81] Wang Y, Balaji V, Kaniyappan S, Krüger L, Irsen S, Tepper K, et al. The release and trans-synaptic transmission of Tau via exosomes. Mol Neurodegener. 2017;12:5. 28086931 10.1186/s13024-016-0143-y PMC5237256

[82] Griflyuk AV, Postnikova TY, Zaitsev AV. Prolonged febrile seizures impair synaptic plasticity and alter developmental pattern of glial fibrillary acidic protein (GFAP)-immunoreactive astrocytes in the hippocampus of young rats. Int J Mol Sci. 2022;23:12224. 36293077 10.3390/ijms232012224 PMC9603570

[83] Xu M, Wang J, Shi J, Wu X, Zhao Q, Shen H, et al. Esketamine mitigates endotoxin-induced hippocampal injury by regulating calcium transient and synaptic plasticity via the NF-α1/CREB pathway. Neuropharmacology. 2025;269:110362. 39947390 10.1016/j.neuropharm.2025.110362

[84] Clarke G, Grenham S, Scully P, Fitzgerald P, Moloney RD, Shanahan F, et al. The microbiome-gut-brain axis during early life regulates the hippocampal serotonergic system in a sex-dependent manner. Mol Psychiatry. 2013;18:666–73. 22688187 10.1038/mp.2012.77

[85] Desbonnet L, Garrett L, Clarke G, Bienenstock J, Dinan TG. The probiotic Bifidobacteria infantis: an assessment of potential antidepressant properties in the rat. J Psychiatr Res. 2008;43:164–74. 18456279 10.1016/j.jpsychires.2008.03.009

[86] Martin-Gallausiaux C, Larraufie P, Jarry A, Béguet-Crespel F, Marinelli L, Ledue F, et al. Butyrate produced by commensal bacteria down-regulates indolamine 2,3-dioxygenase 1 (IDO-1) expression via a dual mechanism in human intestinal epithelial cells. Front Immunol. 2018;9:2838. 30619249 10.3389/fimmu.2018.02838 PMC6297836

[87] Wang D, Li D, Zhang Y, Chen J, Zhang Y, Liao C, et al. Functional metabolomics reveal the role of AHR/GPR35 mediated kynurenic acid gradient sensing in chemotherapy-induced intestinal damage. Acta Pharm Sin B. 2021;11:763–80. 33777681 10.1016/j.apsb.2020.07.017 PMC7982426

[88] Barnes SE, Zera KA, Ivison GT, Buckwalter MS, Engleman EG. Brain profiling in murine colitis and human epilepsy reveals neutrophils and TNFα as mediators of neuronal hyperexcitability. J Neuroinflammation. 2021;18:199. 34511110 10.1186/s12974-021-02262-4 PMC8436533

[89] Kebede V, Ravizza T, Balosso S, Di Sapia R, Canali L, Soldi S, et al. Early treatment with rifaximin during epileptogenesis reverses gut alterations and reduces seizure duration in a mouse model of acquired epilepsy. Brain Behav Immun. 2024;119:363–80. 38608741 10.1016/j.bbi.2024.04.007

[90] Stone TW, Williams RO. Modulation of T cells by tryptophan metabolites in the kynurenine pathway. Trends Pharmacol Sci. 2023;44:442–56. 37248103 10.1016/j.tips.2023.04.006

[91] Okamoto Y, Shikano S. Emerging roles of a chemoattractant receptor GPR15 and ligands in pathophysiology. Front Immunol. 2023;14:1179456. 37457732 10.3389/fimmu.2023.1179456 PMC10348422

[92] Spielbauer J, Glotfelty EJ, Sarlus H, Harris RA, Diaz Heijtz R, Karlsson TE. Bacterial peptidoglycan signalling in microglia: activation by MDP via the NF-κB/MAPK pathway. Brain Behav Immun. 2024;121:43–55. 38971207 10.1016/j.bbi.2024.06.027

[93] Lin Y, Lang H, Gao P, Miao X, Guo Q, Hao Y, et al. Electromagnetic pulse exposure induces neuroinflammation and blood-brain barrier disruption by activating the NLRP3 inflammasome/NF-κB signaling pathway in mice. Ecotoxicol Environ Saf. 2025;292:117972. 40020384 10.1016/j.ecoenv.2025.117972

[94] Bucher V, Herrock OT, Schell S, Visser J, Imberg H, Burke J, et al. Blood-brain barrier injury and neuroinflammation in pre-eclampsia and eclampsia. EBioMedicine. 2025;116:105742. 40344719 10.1016/j.ebiom.2025.105742 PMC12136835

[95] Egerod KL, Petersen N, Timshel PN, Rekling JC, Wang Y, Liu Q, et al. Profiling of G protein-coupled receptors in vagal afferents reveals novel gut-to-brain sensing mechanisms. Mol Metab. 2018;12:62–75. 29673577 10.1016/j.molmet.2018.03.016 PMC6001940

[96] Zhu J, Xu C, Zhang X, Qiao L, Wang X, Yan X, et al. The effect of vagal nerve stimulation on hippocampal-thalamic functional connectivity in epilepsy patients. Brain Res Bull. 2020;163:143–9. 32745494 10.1016/j.brainresbull.2020.07.023

[97] Sawchenko PE. Central connections of the sensory and motor nuclei of the vagus nerve. J Auton Nerv Syst. 1983;9:13–26. 6319474 10.1016/0165-1838(83)90129-7

[98] Zhu J, Xu C, Zhang X, Qiao L, Wang X, Zhang X, et al. Altered amplitude of low-frequency fluctuations and regional homogeneity in drug-resistant epilepsy patients with vagal nerve stimulators under different current intensity. CNS Neurosci Ther. 2021;27:320–9. 32965801 10.1111/cns.13449 PMC7871792

[99] Meng T, Zhang Y, Fu S, Ma S. Gpr35 expression mitigates neuroinflammation and enriches gut *Lactobacillus* to relieve Parkinson’s disease. Research. 2025;8:0846. 40862055 10.34133/research.0846 PMC12376290

[100] Xi Y, Li H, Yu M, Li X, Li Y, Hui B, et al. Protective effects of chlorogenic acid on trimethyltin chloride-induced neurobehavioral dysfunctions in mice relying on the gut microbiota. Food Funct. 2022;13:1535–50. 35072194 10.1039/d1fo03334d

[101] Żarnowska I, Wróbel-Dudzińska D, Tulidowicz-Bielak M, Kocki T, Mitosek-Szewczyk K, Gasior M, et al. Changes in tryptophan and kynurenine pathway metabolites in the blood of children treated with ketogenic diet for refractory epilepsy. Seizure. 2019;69:265–72. 31129366 10.1016/j.seizure.2019.05.006

[102] Yan J, Kothur K, Innes EA, Han VX, Jones HF, Patel S, et al. Decreased cerebrospinal fluid kynurenic acid in epileptic spasms: a biomarker of response to corticosteroids. EBioMedicine. 2022;84:104280. 36174397 10.1016/j.ebiom.2022.104280 PMC9515432

[103] Hanin A, Chollet C, Demeret S, Di Meglio L, Castelli F, Navarro V. Metabolomic changes in adults with status epilepticus: a human case-control study. Epilepsia. 2024;65:929–43. 38339978 10.1111/epi.17899

[104] Yan J, Kothur K, Mohammad S, Chung J, Patel S, Jones HF, et al. CSF neopterin, quinolinic acid and kynurenine/tryptophan ratio are biomarkers of active neuroinflammation. EBioMedicine. 2023;91:104589. 37119734 10.1016/j.ebiom.2023.104589 PMC10165192

[105] Peng Y, Chiu ATG, Li VWY, Zhang X, Yeung WL, Chan SHS, et al. The role of the gut-microbiome-brain axis in metabolic remodeling amongst children with cerebral palsy and epilepsy. Front Neurol. 2023;14:1109469. 36923492 10.3389/fneur.2023.1109469 PMC10009533

[106] Majoie HJM, Rijkers K, Berfelo MW, Hulsman J, Myint A, Schwarz M, et al. Vagus nerve stimulation in refractory epilepsy: effects on pro- and anti-inflammatory cytokines in peripheral blood. Neuroimmunomodulation. 2011;18:52–6. 20639683 10.1159/000315530

[107] Wan L, Shi X, Yan H, Liang Y, Liu X, Zhu G, et al. Abnormalities in *Clostridioides* and related metabolites before ACTH treatment may be associated with its efficacy in patients with infantile epileptic spasm syndrome. CNS Neurosci Ther. 2024;30:e14398. 37553527 10.1111/cns.14398 PMC10805391

[108] Klinkenberg S, van den Borne CJH, Aalbers MW, Verschuure P, Kessels AG, Leenen L, et al. The effects of vagus nerve stimulation on tryptophan metabolites in children with intractable epilepsy. Epilepsy Behav. 2014;37:133–8. 25022821 10.1016/j.yebeh.2014.06.001

[109] Qiao CM, Ma XY, Tan LL, Xia YM, Li T, Wu J, et al. Indoleamine 2, 3-dioxygenase 1 inhibition mediates the therapeutic effects in Parkinson’s disease mice by modulating inflammation and neurogenesis in a gut microbiota dependent manner. Exp Neurol. 2025;385:115142. 39793693 10.1016/j.expneurol.2025.115142

[110] Aslamkhan AG, Xu Q, Loughlin A, Vu H, Pacchione S, Bhatt B, et al. Characterization of indoleamine-2,3-dioxygenase 1, tryptophan-2,3-dioxygenase, and Ido1/Tdo2 knockout mice. Toxicol Appl Pharmacol. 2020;406:115216. 32871117 10.1016/j.taap.2020.115216

[111] Samriti N, Kaur A, Kaur A, Goel RK. Ameliorative effect of diclofenac in rotenone corneal kindling model of drug-resistant epilepsy: edge of dual COX and KMO inhibition. Brain Res. 2025;1846:149246. 39304107 10.1016/j.brainres.2024.149246

[112] Xu J, Huang Y, Wei L, Kong Z, Fu J, Cai L. Kmo inhibition improves seizures and depressive-like behaviors without aggravating cognitive impairment in epileptic mice. Curr Issues Mol Biol. 2025;47:705. 41020827 10.3390/cimb47090705 PMC12468293

[113] Dumont KD, Jannig PR, Porsmyr-Palmertz M, Ruas JL. Constitutive loss of kynurenine-3-monooxygenase changes circulating kynurenine metabolites without affecting systemic energy metabolism. Am J Physiol Endocrinol Metab. 2025;328:E274-85. 39805032 10.1152/ajpendo.00386.2024

[114] Maddison DC, Alfonso-Núñez M, Swaih AM, Breda C, Campesan S, Allcock N, et al. A novel role for kynurenine 3-monooxygenase in mitochondrial dynamics. PLoS Genet. 2020;16:e1009129. 33170836 10.1371/journal.pgen.1009129 PMC7654755

[115] Dircio-Bautista M, Colín-González AL, Aguilera G, Maya-López M, Villeda-Hernández J, Galván-Arzate S, et al. The antiepileptic drug levetiracetam protects against quinolinic acid-induced toxicity in the rat striatum. Neurotox Res. 2018;33:837–45. 29124680 10.1007/s12640-017-9836-4

[116] Fukuyama K, Okada M. Effects of levetiracetam on astroglial release of kynurenine-pathway metabolites. Br J Pharmacol. 2018;175:4253–65. 30153331 10.1111/bph.14491 PMC6193875

[117] Fazio F, Lionetto L, Molinaro G, Bertrand HO, Acher F, Ngomba RT, et al. Cinnabarinic acid, an endogenous metabolite of the kynurenine pathway, activates type 4 metabotropic glutamate receptors. Mol Pharmacol. 2012;81:643–56. 22311707 10.1124/mol.111.074765

[118] Ngomba RT, Ferraguti F, Badura A, Citraro R, Santolini I, Battaglia G, et al. Positive allosteric modulation of metabotropic glutamate 4 (mGlu4) receptors enhances spontaneous and evoked absence seizures. Neuropharmacology. 2008;54:344–54. 18022649 10.1016/j.neuropharm.2007.10.004

[119] Singh T, Kaur T, Goel RK. Adjuvant quercetin therapy for combined treatment of epilepsy and comorbid depression. Neurochem Int. 2017;104:27–33. 28065794 10.1016/j.neuint.2016.12.023

[120] Singh T, Kaur T, Goel RK. Ferulic acid supplementation for management of depression in epilepsy. Neurochem Res. 2017;42:2940–8. 28608235 10.1007/s11064-017-2325-6

[121] Kocki T, Kocki J, Wielosz M, Turski WA, Urbanska EM. Carbamazepine enhances brain production of kynurenic acid *in vitro*. Eur J Pharmacol. 2004;498:325–6. 15364012 10.1016/j.ejphar.2004.07.088

[122] Kocki T, Wielosz M, Turski WA, Urbanska EM. Enhancement of brain kynurenic acid production by anticonvulsants–novel mechanism of antiepileptic activity? Eur J Pharmacol. 2006;541:147–51. 16765940 10.1016/j.ejphar.2006.05.015

[123] Ghosh C, Marchi N, Hossain M, Rasmussen P, Alexopoulos AV, Gonzalez-Martinez J, et al. A pro-convulsive carbamazepine metabolite: quinolinic acid in drug resistant epileptic human brain. Neurobiol Dis. 2012;46:692–700. 22426401 10.1016/j.nbd.2012.03.010 PMC4001854

[124] Vezzani A, Wu HQ, Angelico P, Stasi MA, Samanin R. Quinolinic acid-induced seizures, but not nerve cell death, are associated with extracellular Ca2+ decrease assessed in the hippocampus by brain dialysis. Brain Res. 1988;454:289–97. 2970276 10.1016/0006-8993(88)90829-3

[125] Vezzani A, Wu HQ, Tullii M, Samanin R. Anticonvulsant drugs effective against human temporal lobe epilepsy prevent seizures but not neurotoxicity induced in rats by quinolinic acid: electroencephalographic, behavioral and histological assessments. J Pharmacol Exp Ther. 1986;239:256–63. 2945004

[126] Kaur A, Kumar S, Goel RK. Adjunct antiseizure effect of clotrimazole in a rotenone corneal kindling mouse model of mitochondrial drug-resistant epilepsy. Epilepsy Res. 2023;198:107246. 37925976 10.1016/j.eplepsyres.2023.107246

[127] Singh T, Goel RK. Evidence in support of using a neurochemistry approach to identify therapy for both epilepsy and associated depression. Epilepsy Behav. 2016;61:248–57. 27423076 10.1016/j.yebeh.2016.05.005

[128] Xie W, Cai L, Yu Y, Gao L, Xiao L, He Q, et al. Activation of brain indoleamine 2,3-dioxygenase contributes to epilepsy-associated depressive-like behavior in rats with chronic temporal lobe epilepsy. J Neuroinflammation. 2014;11:41. 24594021 10.1186/1742-2094-11-41 PMC3975854

[129] Singh T, Goel RK. Adjuvant indoleamine 2,3-dioxygenase enzyme inhibition for comprehensive management of epilepsy and comorbid depression. Eur J Pharmacol. 2016;784:111–20. 27189423 10.1016/j.ejphar.2016.05.019

[130] Singh T, Bagga N, Kaur A, Kaur N, Gawande DY, Goel RK. Agmatine for combined treatment of epilepsy, depression and cognitive impairment in chronic epileptic animals. Biomed Pharmacother. 2017;92:720–5. 28586743 10.1016/j.biopha.2017.05.085

[131] Agarwal S, Vyas P, Nirwan N, Vohora D. Effect of lacosamide on neuroinflammation-mediated seizures comorbid with depression in C57BL/6 mice- role of kynurenine pathway. Epilepsy & Behavior: E&B. 2021;123:108262. 10.1016/j.yebeh.2021.108262 34425328

